# The Metabolome of a Cyanobacterial Bloom Visualized by MS/MS-Based Molecular Networking Reveals New Neurotoxic Smenamide Analogs (C, D, and E)

**DOI:** 10.3389/fchem.2018.00316

**Published:** 2018-07-26

**Authors:** Christopher W. Via, Evgenia Glukhov, Samuel Costa, Paul V. Zimba, Peter D. R. Moeller, William H. Gerwick, Matthew J. Bertin

**Affiliations:** ^1^Department of Biomedical and Pharmaceutical Sciences, College of Pharmacy, University of Rhode Island, Kingston, RI, United States; ^2^Center for Marine Biotechnology and Biomedicine, Skaggs School of Pharmacy and Pharmaceutical Sciences, Scripps Institution of Oceanography, University of California at San Diego, La Jolla, CA, United States; ^3^Center for Coastal Studies and Department of Life Sciences, Texas A&M Corpus Christi, Corpus Christi, TX, United States; ^4^Emerging Toxins Program, Hollings Marine Laboratory, National Ocean Service/NOAA, Charleston, SC, United States

**Keywords:** *Trichodesmium*, molecular networking, cyanotoxins, harmful algal blooms, metabolomics

## Abstract

Members of the cyanobacterial genus *Trichodesmium* are well known for their substantial impact on nitrogen influx in ocean ecosystems and the enormous surface blooms they form in tropical and subtropical locations. However, the secondary metabolite composition of these complex environmental bloom events is not well known, nor the possibility of the production of potent toxins that have been observed in other bloom-forming marine and freshwater cyanobacteria species. In the present work, we aimed to characterize the metabolome of a *Trichodesmium* bloom utilizing MS/MS-based molecular networking. Furthermore, we integrated cytotoxicity assays in order to identify and ultimately isolate potential cyanotoxins from the bloom. These efforts led to the isolation and identification of several members of the smenamide family, including three new smenamide analogs (**1–3**) as well as the previously reported smenothiazole A-hybrid polyketide-peptide compounds. Two of these new smenamides possessed cytotoxicity to neuro-2A cells (**1** and **3**) and their presence elicits further questions as to their potential ecological roles. HPLC profiling and molecular networking of chromatography fractions from the bloom revealed an elaborate secondary metabolome, generating hypotheses with respect to the environmental role of these metabolites and the consistency of this chemical composition across genera, space and time.

## Introduction

Blooms of toxin-producing cyanobacteria (harmful algal blooms, HABs) continue to be a threat to water resources in the U.S. and across the globe (Carmichael and Boyer, 2016). Research surrounding these bloom events with respect to cyanobacteria has generally focused on freshwater planktonic species and a suite of well-characterized toxins, including the anatoxins, saxitoxins and microcystins (Bláha et al., 2009). However, species of cyanobacteria in the marine realm have been a prolific source of exquisitely potent cytotoxic metabolites (Luesch et al., 2001; Taori et al., 2008; Pereira et al., 2012). Members of the bloom-forming genus *Trichodesmium* are an understudied group of marine cyanobacteria with respect to toxin production and environmental impact. With respect to new natural products, the cyclic peptide trichamide was characterized from a cultured strain of *Trichodesmium erythraeum*, although no significant cytotoxicity was observed against HCT-116 cells and CEM-TART cells when tested at 10 and 50 μg/mL, respectively (Sudek et al., 2006). The lipoamides, credneramides A and B were isolated and characterized from a field-collected benthic cyanobacterium identified as a new species of *Trichodesmium* (Malloy et al., 2012). These metabolites inhibited spontaneous calcium oscillations in murine cerebrocortical neurons (Malloy et al., 2012). Several known cyanotoxins, such as anatoxin, saxitoxin, microcystins and aplysiatoxins have been reported from *Trichodesmium* blooms collected from distinct geographic areas (Ramos et al., 2005; Detoni et al., 2016; Shunmugam et al., 2017).

**Figure 1:**
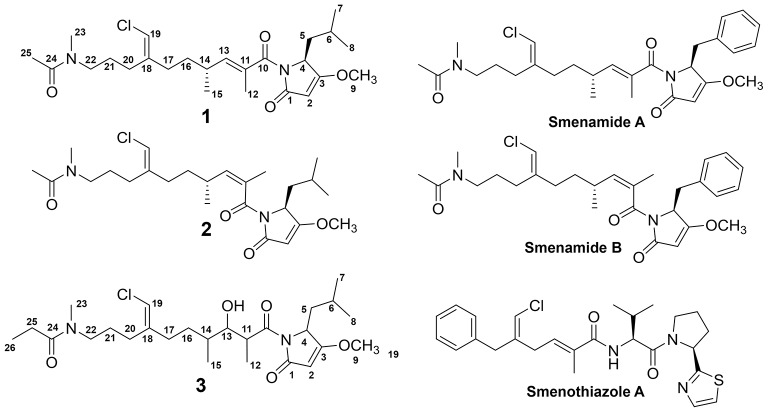
Structures of **1-3**, smenamides A and B and smenothiazole A.

In the current report, we detail the comprehensive metabolic profiling of a *Trichodesmium* bloom collected from the western Gulf of Mexico utilizing MS/MS-based molecular networking (Watrous et al., 2012; Yang et al., 2013; Wang et al., 2016) and cytotoxicity assays. In our previous work on this *Trichodesmium* bloom, we have utilized cytotoxicity assays, NMR-guided isolation and MS-guided isolation independently to characterize chlorinated polyketides and hybrid polyketide peptides (Bertin et al., 2016, 2017a,b; Belisle et al., 2017). The current report attempts to describe the *Trichodesmium* bloom metabolome more completely, focusing on a networking tool to cluster molecules based on similarities in the MS/MS fragmentation patterns (Watrous et al., 2012). Our efforts ultimately led to the isolation and characterization of three new members of the smenamide family of molecules (**1–3**) and the previously reported smenothiazole A (Figure 1). Smenamides C and E demonstrated potent neurotoxicity (**1** and **3**).

## Materials and methods

### General experimental procedures

Optical rotations were measured using a Jasco P-2000 polarimeter. UV spectra were measured using a Beckman Coulter DU-800 spectrophotometer. CD spectra were recorded using a Jasco J-1100 CD spectrometer. NMR spectra were collected using a Bruker 800 MHz NMR instrument. Additional NMR spectra were recorded on a Varian 500 MHz NMR instrument. HRESIMS analysis was performed using an AB SCIEX TripleTOF 4600 mass spectrometer with Analyst TF software. LC-MS/MS analysis was carried out using a ThermoFinnigan LCQ AdvantageMax mass spectrometer with an electrospray ionization (ESI) source. Semi-preparative HPLC was carried out using a Dionex UltiMate 3000 HPLC system and Agilent 1100 series system each equipped with a micro vacuum degasser, an autosampler and a diode-array detector.

### Collection, extraction and fractionation of bloom material

Samples from a localized bloom of *Trichodesmium* were collected from Padre Island, Corpus Christi, TX during 9–11 May 2014 as described previously (Bertin et al., 2016, 2017a,b; Belisle et al., 2017). Briefly, bloom material was collected in 5-gallon buckets from *ca*. 0.5-meter water depth and concentrated by gentle filtration through an 18 μm mesh screen. A subsample of the cell mass was examined microscopically and identified using Komárek and Anagnostidis (2005) as being dominated by cyanobacteria of the genus *Trichodesmium*. The material was frozen and shipped for further chemical analysis. The biomass (ca. 14 g dry weight) was repeatedly extracted with 2:1 CH_2_Cl_2_:CH_3_OH and the extracts were combined and evaporated under reduced pressure (3.95 g). The extract was reconstituted in hexanes and applied to silica gel (300 mL) in a wide fritted column with a vacuum attachment. The extract was fractionated using a stepped gradient from 100% hexanes to 100% CH_3_OH resulting in nine fractions. Seven of the nine fractions (C-I) were further analyzed by means of cytotoxicity assays and MS/MS-based molecular networking. The first two fractions: 100% hexanes (A) and 90% hexanes in EtOAc (B) were intended to remove hydrocarbons and exceedingly lipophilic substances from the sample and were not analyzed further.

### Molecular networking

Fractions C-I were subjected to LC-MS/MS analysis with data collection in data-dependent acquisition mode on a ThermoFinnigan LCQ AdvantageMax mass spectrometer with an electrospray ionization (ESI) source. A Kinetex 5 μm C18 column (100 × 4.6 mm) was used for separation of analytes. The LC method consisted of a linear gradient from 30 to 99% CH_3_CN in water + 0.1% formic acid over 17 minutes, followed by an isocratic period at 99% CH_3_CN of 3 minutes. The flow rate was held at 0.6 mL/min. The MS spray voltage was 5 kV with a capillary temperature of 400°C. For the MS/MS component, the CID isolation width was 2.0 and the collision energy was 35.0 eV. The raw data files were converted to mzXML format using MSConvert from the ProteoWizard suite (http://proteowizard.sourceforge.net/tools.shtml)^1^. The molecular network was generated using the online platform at Global Natural Products Social Molecular Networking website (gnps.ucsd.edu) using parameters detailed in Table S5. The network was visualized using the Browser Network Visualizer tool available on the gnps website.

### Isolation of 1–3 and smenothiazole A

Fractions G (100% EtOAc, 104.0 mg) and H (75% EtOAc in CH_3_OH, 286.9 mg) were chosen for further purification based on the quantity of network ions in these fractions, the molecular features of these ions (ratio of M^+^ and M^+2^ isotope), and cytotoxicity results of the mixed chromatography fractions. Fractions G and H were combined based on similarities in LC-MS/MS profiles and ^1^H NMR resonances. The combined sample was further fractionated over a 2 g C18 SPE column eluting with 50% water in CH_3_CN (13.7 mg), 100% CH_3_CN (143.2 mg), 100% CH_3_OH (74.5 mg) and 100% EtOAc (54.5 mg). The fraction eluting with 100% CH_3_CN was subjected to reversed phase HPLC using a YMC 5 μm ODS column (250 × 10 mm); mobile phase: 65% CH_3_CN /35% water with 0.05% formic acid added to each solvent, flow 3 mL/min. Fractions were collected based on UV characteristics and HPLC fractions were analyzed by HRESIMS for ions of interest from the molecular network. Further purification was carried out using the YMC column mentioned above; mobile phase: 80% CH_3_CN in water with 0.05% formic acid added to each solvent, flow 3 mL/min resulted in the isolation of 7.0 mg of **1** (t_R_, 11.5 min). A mobile phase of 65% CH_3_CN in water with 0.05% formic acid added to each solvent, flow 3 mL/min was used to isolate 0.6 mg of **2** (t_R_, 26.0 min) and 0.3 mg of **3** (t_R_, 21.2 min). A final purification was carried out using a YMC 5 μm ODS column (250 × 10 mm); mobile phase: 80% CH_3_CN in water with 0.1% formic acid added to each solvent, flow 3 mL/min and 2.0 mg of smenothiazole A was isolated (t_R_, 5.0 min).

*Smenamide C* (**1**): colorless oil; [α]^25^_D_ +38.2 (*c* 0.20, CH_3_OH); UV (CH_3_OH) λ_max_ (log ε) 203 (4.2), 238 (4.0) nm; ^1^H NMR (800 MHz, DMSO-*d*_6_) and ^13^C NMR (200 MHz, DMSO-*d*_6_), see Table 1; HRESIMS *m/z* 467.2661 [M+H]^+^ (calcd for C_25_H_40_N_2_O_4_Cl, 467.2677) and *m/z* 489.2486 [M+Na]^+^ (calcd for C_25_H_39_N_2_O_4_ClNa, 489.2496).

**Table 1:** NMR data for Smenamide C (**1**) *Z*-conformer (800 MHz for ^1^H NMR; 200 MHz for ^13^C NMR, DMSO).

**Position**	**δ_C_, type**	**δ_H_ (*J* in Hz)**	**HMBC**	**COSY**
1	169.3, qC			
2	93.7, CH	5.28, s	1, 4, 10	
3	180.3, qC			
4	57.6, CH	4.75, t (5.3)	1, 2, 3, 5, 6, 10	5a, 5b
5a	38.6, CH_2_	1.72, m	3, 4, 7, 8	4, 5b
5b		1.59, m	3, 4, 7, 8	4, 5a
6	24.2, CH	1.66, m	4, 5, 7, 8	7, 8
7	24.0, CH_3_	0.85, d (6.6)	5, 8	6
8	23.3, CH_3_	0.87, d (6.6)	5, 7	6
9	59.7, CH_3_	3.87, s	3	
10	170.6, qC			
11	131.7, qC			
12	13.9, CH_3_	1.77, s	10, 11, 13	
13	141.9, CH	5.59, d (9.8)	10, 11, 12, 14, 15, 16	14
14	32.2, CH	2.45, m	11, 13, 15, 16, 17	13, 15, 16a, 16b
15	20.4, CH_3_	0.94, d (6.6)	14, 16, 18	14
16a	34.9, CH_2_	1.49, m	17, 18	14, 17a, 17b
16b		1.35, m	17, 18	14, 17a, 17b
17a	32.1, CH_2_	2.22, m	14, 16, 18, 19, 20	16a, 16b, 17b
17b		2.10, ovlp^a^	14, 16, 18, 19, 20	16a, 16b, 17a
18	142.8, qC			
19	112.6, CH	6.04, s	17, 18, 20	
20a	27.4, CH_2_	2.12, m	17, 18, 19, 21, 22	21
20b		2.07, ovlp	17, 18, 19, 21, 22	21
21	24.8, CH_2_	1.56, m	20, 22	20a, 20b, 22
22	46.7, CH_2_	3.26, m	20, 21, 23, 24	21
23	35.9. CH_3_	2.94, s	22, 24	
24	169.9, qC			
25	22.1, CH_3_	1.97, s	23, 24	

a *Overlapping signals*.

*Smenamide D* (**2**): colorless oil; [α]^25^_D_ +16.8 (*c* 0.10, CH_3_OH); UV (CH_3_OH) λ_max_ (log ε) 203 (3.4), 240 (3.3) nm; ^1^H NMR (800 MHz, CDCl_3_) and ^13^C NMR (200 MHz, CDCl_3_), see Table S3; HRESIMS *m/z* 467.2693 [M+H]^+^ (calcd for C_25_H_40_N_2_O_4_Cl, 467.2677) and *m/z* 489.2492 [M+Na]^+^ (calcd for C_25_H_39_N_2_O_4_ClNa, 489.2496).

*Smenamide E* (**3**): colorless oil; [α]^25^_D_ +21.9 (*c* 0.05, CH_3_OH); UV (CH_3_OH) λ_max_ (log ε) 203 (4.1), 235 (3.8) nm; ^1^H NMR (800 MHz, DMSO-*d*_6_) and ^13^C NMR (200 MHz, DMSO-*d*_6_), see Table 2; HRESIMS *m/z* 499.2935 [M+H]^+^ (calcd for C_26_H_44_N_2_O_5_Cl, 499.2939).

**Table 2:** NMR data for Smenamide E *Z/E*-conformers^a^ (**3**) (800 MHz for ^1^H NMR; 200 MHz for ^13^C NMR, DMSO).

**Position**	**δ_C_, type**	**δ_H_ (*J* in Hz)**	**HMBC**	**COSY**
1	170.3, qC			
2	94.2, CH	5.31, s	1, 3, 4	
3	180.8, qC			
4	58.1, CH	4.63, m	1, 2, 3, 5, 6, 10	5
5	39.2, CH_2_	1.72, m	3, 4, 6, 7, 8	4
6	24.0, CH	1.76, m	4, 5, 7, 8	7, 8
7	24.3, CH_3_	0.84, d	5, 6, 8	6
8	22.9, CH_3_	0.86, d	5, 6, 7	6
9	59.5, CH_3_	3.86, s	3	
10	175.8, qC			
11	42.7, CH	3.95, m	10, 12, 13	12, 13
12	14.4, CH_3_	0.90, d (6.8)	10, 11, 13	11
13	74.3, CH	3.73, m	10, 11, 15	11, OH-13
OH-13		4.40, m	11, 13, 14	13
14	34.3, CH	1.48, ovlp^b^	13, 15	15
15	12.9, CH_3_	0.81, d (6.2)	13, 14, 16	14
16a	31.5, CH_2_	1.48, ovlp	13, 14, 15, 17	16b, 17a
16b		1.26, m	13, 14, 15, 17	16a, 17b
17a	28.1, CH_2_	2.25, m	16, 18, 19	16a, 16b
17b		2.13, m	16, 18, 19	16a, 16b
18	143.0 [142.8], qC			
19	112.3 [112.6], CH	6.04 [6.06], s	17, 18, 20	20
20	31.6 [31.4], CH_2_	2.02 [2.07], t (7.5)	18, 19, 21, 22	21
21	25.4 [26.5], CH_2_	1.55 [1.63], m	20, 22	20, 22
22	46.9 [48.9], CH_2_	3.24 [3.23], m	20, 21, 23, 24	21
23	35.1 [33.2], CH_3_	2.94 [2.79], s	22, 24	
24	173.0 [172.7], qC			
25	26.3 [25.7], CH_2_	2.27 [2.29], m	24, 26	26
26	9.7 [10.1], CH_3_	0.97 [0.98], t (7.4)	24, 25	25

a *E-conformer NMR values of **3** in brackets*.

b *Overlapping signals*.

*Smenothiazole A*: colorless oil; [α]^23^_D_−5.8 (*c* 0.10, CH_3_OH) UV (CH_3_OH) λ_max_ (log ε) 202 (3.4) nm; ^1^H NMR (800 MHz, DMSO-*d*_6_) and ^13^C NMR (200 MHz, DMSO-*d*_6_), see Table S4; HRESIMS *m/z* 486.1984 [M+H]^+^ (calcd for C_26_H_33_ClN_3_O_2_S, 486.1982).

### Cytotoxicity assay

Neuro-2A cells and HCT-116 cells were added to assay plates in 100 μl of Eagle's Minimum Essential Media (EMEM) or 100 μl of McCoy's 5A media respectively each supplemented with 10% FBS at a density of 5,000 cells/well. Cells were incubated overnight (37°C, 5% CO_2_) and examined microscopically to confirm confluence and adherence. Mixed chromatography fractions (C-I) were dissolved in DMSO (1% v/v) and tested at concentrations of 40 and 4 μg/mL with 10 μM doxorubicin used as a positive control. Compounds **1–3** were dissolved in DMSO (1% v/v) and added to the cells in the range of 100 to 0.1 μM in order to generate EC_50_ curves. Four technical replicates were prepared for each concentration and each assay was performed in triplicate. Doxorubicin was used as a positive control (EC_50_: 0.043 ± 0.032 μM for neuro-2A cells; EC_50_: 0.071 ± 0.004 μM for HCT-116 cells) and DMSO (1% v/v) was used as a negative control. Assays were resolved as previously reported (Bertin et al., 2017b) and EC_50_ curves were generated using Graphpad Prism software.

## Results

### *trichodesmium* bloom-cytotoxicity of chromatography fractions and molecular network

**Figure 2:**
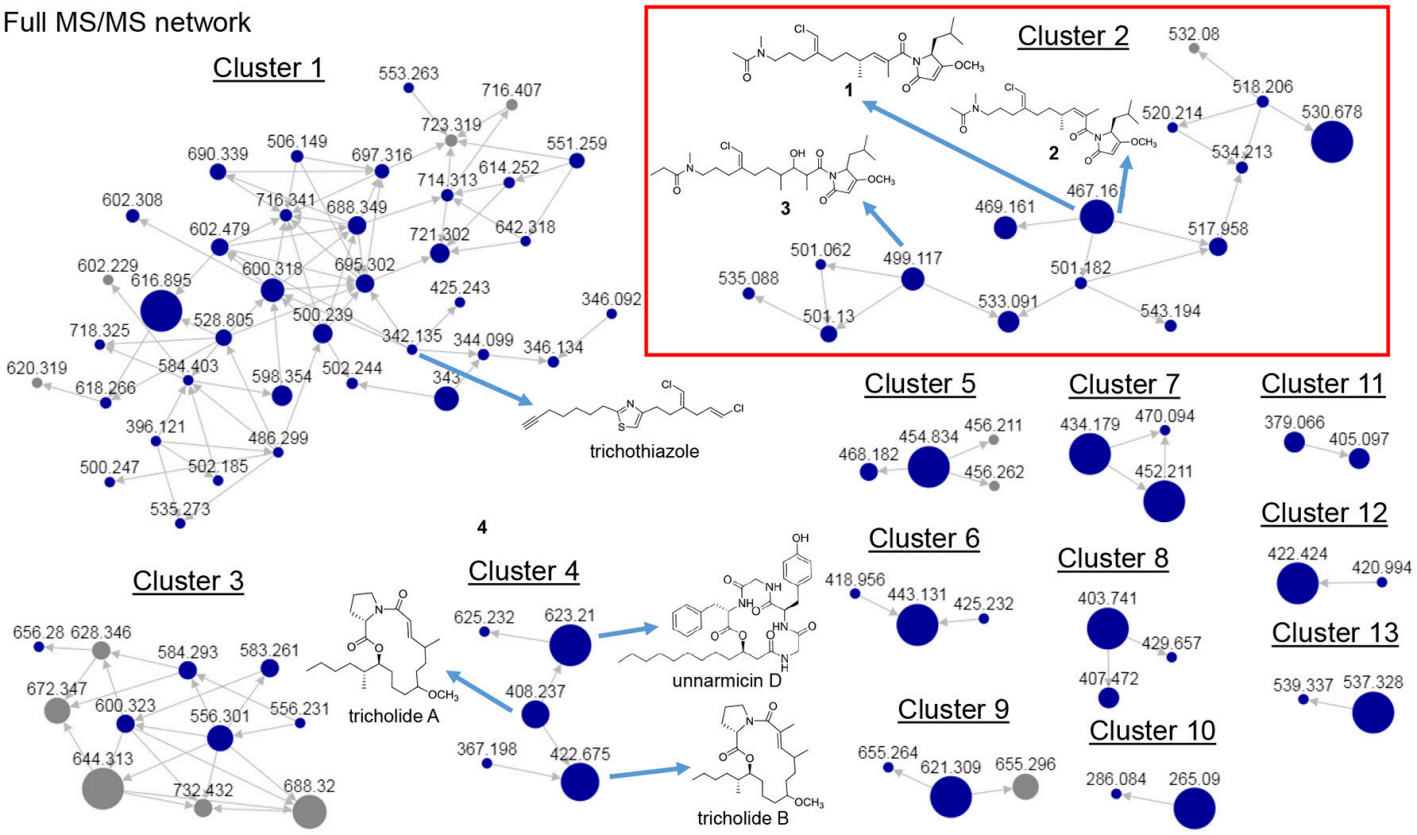
Full MS/MS-based molecular network of *Trichodesmium* bloom. Previously identified molecules trichothiazole, tricholides A and B and unnarmicin D are noted. Cluster 2 (red box) shows new metabolites **1-3** (*m/z* 467.161, 467.161 and 499.117, respectively).

**Figure 3:**
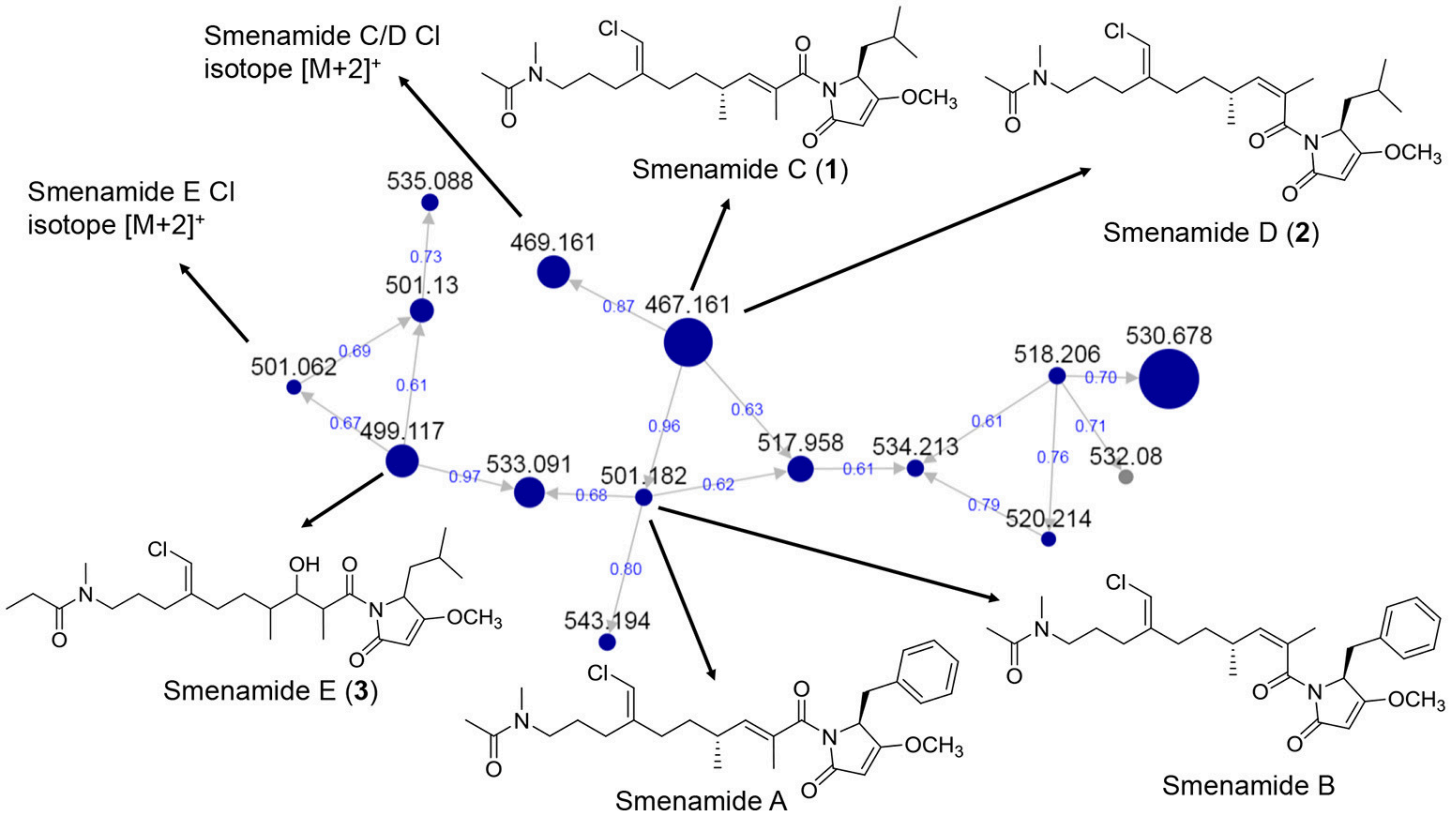
MS/MS-based molecular networking cluster identifying **1–3** and smenamides A or B. Nodes are labeled with parent *m/z* values. Edges are labeled with cosine scores. Node size is relative to ion count.

Several of the chromatography fractions (D–H) derived from the bloom material showed strong cytotoxicity against neuro-2A cells at 40 μg/mL (Figure S1). Fraction D showed the greatest potency at 4 μg/mL. Examination of the molecular network showed that compounds from cluster 3 were major ions in fraction D (Figure 2). However, these metabolites were not isolable following further purification procedures. The majority of the metabolites in the molecular network were found in fractions F–I. We identified Cluster 2 as a molecular cluster of interest due to the number of ions in the cluster and the M^+^ and M^+2^ ratio indicating a single chlorine atom in each of these metabolites (cf. Figures 2, 3). Additionally, fractions G and H showed potent cytotoxicity against neuro-2A cells; thus, our subsequent purification efforts centered on these two fractions. HPLC analysis indicated abundant metabolites in the combined G+H HPLC pre-fraction (Figure S2) and repeated chromatography resulted in the isolation of **1–3** as optically active colorless oils.

### Structure characterization of 1–3

**Figure 4:**
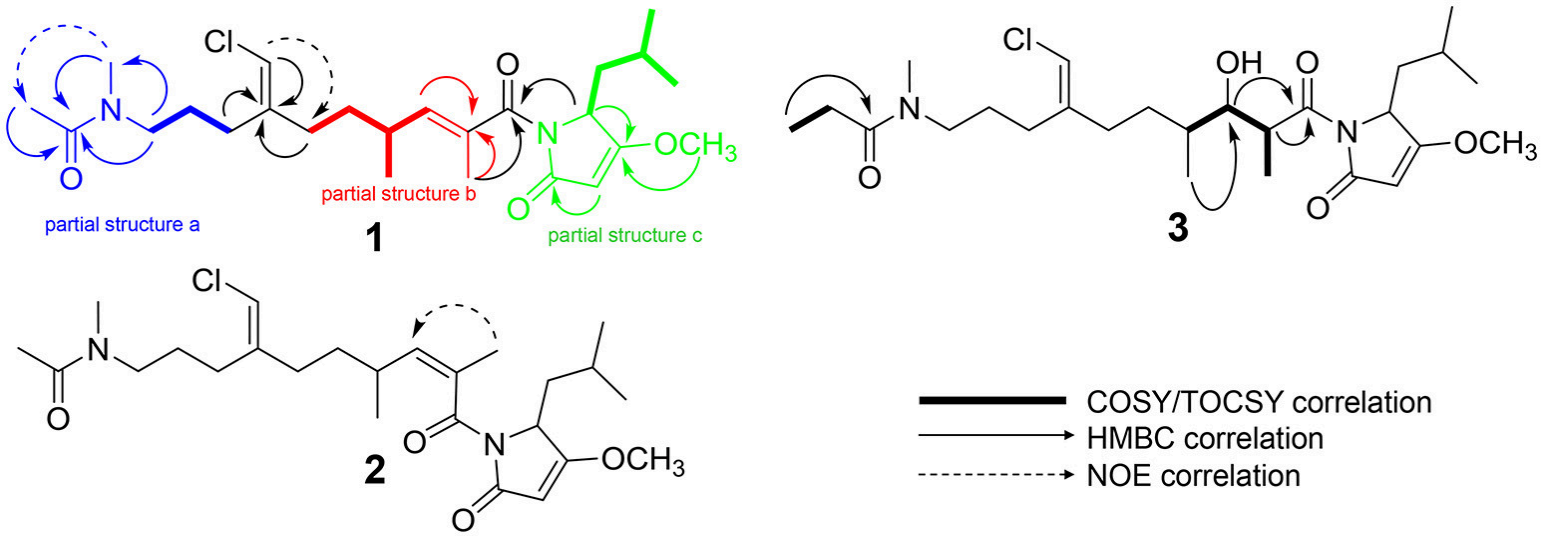
Select 2D NMR correlations of compounds **1–3**.

HRESIMS analysis of **1** gave an [M+H]^+^ of *m/z* 467.2661, suggesting a molecular formula of C_25_H_39_N_2_O_4_Cl and a requirement of 7 degrees of unsaturation. Examination of the ^1^H NMR spectrum of **1** showed several resonances with split signals in a 1:1 ratio, a phenomenon observed in several cyanobacteria metabolites with methylated tertiary amides such as smenamides A and B and kalkitoxin (Wu et al., 2000; Teta et al., 2013). The split signals were determined to be the result of two conformers in the *E* and *Z* configuration at the tertiary amide functionality in **1**. This phenomenon was observed for all three new metabolites (**1–3**); in the structure characterization for each of these compounds, the data for the *Z* conformer is discussed. NMR data tables in the Supporting Information provide information on the *E* conformer. While the multiple conformers presented difficulties in NMR interpretation, three partial structures of **1** (a–c) were characterized initially based on ^1^H-^1^H COSY spin systems followed by HMBC correlation analysis (Figure 4). In the first partial structure (a), a moderately deshielded diastereotopic methylene group (H-20a, δ_H_ 2.12; H-20b, δ_H_ 2.07) was correlated by COSY to a second methylene group (H_2_-21, δ_H_ 1.56) which itself was correlated by COSY to a third methylene group (H_2_-22, δ_H_ 3.26). This latter deshielded methylene was correlated by HMBC to C-23 (δ_C_ 35.9) and the C-24 carbonyl (δ_C_ 169.9). The singlet methyl (H_3_-25, δ_H_ 1.97) showed an HMBC correlation to C-24 and characterized the western half of **1** with an *N*-methyl acetamide functionality. In the second partial structure of **1** (b), another moderately deshielded diastereotopic methylene group (H-17a, δ_H_ 2.22; H-17b, δ_H_ 2.10) showed COSY correlations to the H-16 methylene (H-16a, δ_H_ 1.49; H-16b, δ_H_ 1.35). H_2_-16 showed COSY correlations to the H-14 methine (δ_H_ 2.45), which itself showed COSY correlations to a doublet methyl (H_3_-15, δ_H_ 0.94) and olefinic proton (H-13, δ_H_ 5.59). A singlet methyl (H_3_-12, δ_H_ 1.77) and H-13 showed HMBC correlations to a quaternary carbon (C-11, δ_C_ 131.7) and the C-10 carbonyl (δ_C_ 170.6) extending the polyketide chain of **1**. The C-11–C-13 olefin was assigned *E* geometry based on the ^13^C chemical shift of C-12 (δ_C_ 13.7) compared to δ_C_ 20.1 for the *Z* geometry (see below). The two sets of moderately deshielded methylenes (H_2_-20 and H_2_-17) showed HMBC correlations to the quaternary carbon at C-18 (δ_C_ 142.8). A deshielded methine singlet (H-19, δ_H_ 6.04) also showed an HMBC correlation to C-18, supporting an exomethylene vinyl chloride bridge connecting partial structures a and b. The configuration of the vinyl chloride was assigned as *Z* based on NOE correlations from H-19 to H_2_-17 and H_2_-16. The chemical shift of C-10 was consistent with that of an amide functionality and COSY correlations from H-4 to H-8 supported the assignment of a leucine residue in the third partial structure. However, the chemical shift at C-3 was somewhat deshielded for that of a standard amide or ester carbonyl (δ_C_ 180.3). An *O*-methyl singlet (H_3_-9, δ_H_ 3.87) was correlated to C-3 by HMBC supporting the presence of a methoxy functionality. Additionally, H-2 (δ_H_ 5.28) was correlated to C-3 and the C-1 carbonyl by HMBC. HMBC correlations from H-2 and H-4 to C-10 connected the third partial structure to the remainder of the molecule, establishing an isobutyl-methoxypyrrolinone moiety and satisfying the final three degrees of unsaturation required by the molecular formula. The structure of **1** was established as a highly functionalized linear polyketide-peptide of the smenamide family (Teta et al., 2013). While the correlations and chemical shifts described above relate to the *Z* conformer of **1**, NMR data for the *E* conformer were also analyzed, and are listed in Table S1.

The absolute configuration of **1** (4*S*, 14*R*) was determined to be identical to that of smenamide A by comparison of the CD spectrum of **1** to that of naturally occurring smenamide A (Caso et al., 2017). The spectra were nearly identical in sign and magnitude.

HRESIMS analysis of **2** gave an [M+H]^+^ of *m/z* 467.2693, suggesting a molecular formula of C_25_H_39_N_2_O_4_Cl, identical to that of **1**. Examination of ^1^H NMR, multiplicity-edited HSQC, and HMBC spectra of **1** and **2** showed that the two molecules were nearly identical (cf. Tables S2 and S3). ^13^C NMR differences were most pronounced at C-12 (δ_C_ 13.7 in **1**; δ_C_ 20.1 in **2**) and C-13 (δ_C_ 142.7 in **1**; δ_C_ 135.9 in **2**). These chemical shifts and the NOE correlations between H_3_-12 and H-13 in **2** supported the *Z* configuration of the C-11–C-13 olefin in **2** and established **2** as a geometric isomer of **1**. The absolute stereochemistry of **2** is proposed to be identical to that of **1** based on similarity in optical rotation values.

HRESIMS analysis of **3** gave an [M+H]^+^ of *m/z* 499.2935, suggesting a molecular formula of C_26_H_43_N_2_O_5_Cl, and a requirement of 6 degrees of unsaturation. The examination of ^1^H and ^13^C NMR data and the placement of *m/z* 499 in the same molecular network cluster as smenamide C and D (**1** and **2**), suggested that **3** was a close structural analog. The reduction in degrees of unsaturation in **3** compared to **1** was due to the presence of a secondary alcohol at C-13 (H-13, δ_H_ 3.73; C-13, δ_C_ 74.3) in **3** and a methine at C-11 (H-11, δ_H_ 3.95; C-11, δ_C_ 42.7). The H-11 and H-13 methine protons were correlated by COSY and H-13 also showed a COSY correlation to H-14 (δ_H_ 1.48). Additionally, the C-25 methyl resonance of the acetyl group in **1** and **2** was not present in **3**. COSY correlations between a methylene at H-25 (δ_H_ 2.27) and a methyl triplet at H-26 (δ_H_ 0.97) supported an *N*-methylpropanamide functionality in **3** and completed the planar structure of smenamide E (**3**). The secondary alcohol of **3** was resistant to acylation with Mosher's acid chloride and the configuration of this position could not be determined by chemical derivative formation. Therefore, in the current report, we report only the planar structure for this new metabolite.

During the attempt to isolate the compound with an *m/z* 530 from Cluster 2 in the network (Figure 3), we isolated a peptidic compound with spectrometric and spectroscopic characteristics consistent with that of the previously reported cytotoxin smenothiazole A (Esposito et al., 2015). Analysis of NMR data, optical rotation value, and CD spectra (negative Cotton effect at 234 nm, Figure S35) confirmed its identity.

### Cytotoxicity of 1–3

Smenamides C and E (**1** and **3**) showed greater cytotoxicity to neuro-2A cells than to the human colon cancer cell line HCT-116. Smenamide E (**3**) showed the greatest potency to neuro-2A cells with an EC_50_ value of 4.8 ± 0.6 μM (EC_50_: 18.6 ± 1.8 μM against HCT-116 cells). Smenamide C (**1**) showed similar selective potency (EC_50_ neuro-2A: 7.2 ± 3.1 μM; EC_50_ HCT-116: 20.9 ± 2.1 μM). Interestingly, smenamide D (**2**), the geometric isomer of smenamide C (**1**), did not show cytotoxicity against either cell line.

## Discussion

In our previous work on *Trichodesmium* blooms and their natural products, we have utilized cytotoxicity assays, NMR-guided isolation, and MS-guided isolation (Bertin et al., 2016, 2017a,b; Belisle et al., 2017). We have previously characterized the cytotoxic polyketide trichophycin A, the polyketides trichotoxins A and B, and the moderately cytotoxic polyketide-peptide trichothiazole (Bertin et al., 2016, 2017b; Belisle et al., 2017). In the current network, we did observe an *m/z* value consistent with trichothiazole (Figure 2). The node was in Cluster 1 (*m/z* 342) and showed an identical MS/MS fragmentation pattern to that of trichothiazole. However, we did not observe nodes for trichophycin A or trichotoxins A and B. It should be noted that the macrocyclic polyketide-peptides tricholides A and B and unnarmicin D (Bertin et al., 2017a) also clustered in the network (Figure 2, Cluster 4). It may be that some metabolites in the bloom metabolome do not ionize well by ESI+ or give informative fragments during MS/MS acquisition; this represents a limitation in the implementation of MS/MS-based networking to describe bloom metabolomes. Thus, the metabolite information gained in the network may be somewhat biased toward peptides and hybrid polyketide-peptides. Nevertheless, taking into account the limitations of this approach, molecular networking was a remarkable tool for visualizing a complex metabolome rich in metabolites with intriguing structural elements and cytotoxicity to neuro-2A cells. Analyzing fractions C-I using the networking procedure identified 93 nodes that were members of 13 clusters. This approach allowed us to isolate and characterize three new members of the smenamide family (**1–3**). Furthermore, within the smenamide cluster, we tentatively identified the known compound smenamide A or B (double bond isomers of each other at *m/z* 501). This later node in Cluster 2 (Figure 3) showed an identical MS/MS fragmentation pattern to that of smenamide A/B from published data and the HRESIMS analysis supported this identification (Figure S36) (Teta et al., 2013). Both of these known metabolites are very potent cytotoxins with IC_50_ values around 50 nM against Calu-1 cells (Teta et al., 2013). Smenamides C and E (**1** and **3**) were less potent cytotoxins than smenamide A and B, possibly due to the replacement of the phenylalanine amino acid unit with leucine (**1**, **3**). Intriguingly, smenamide D (**2**) was not cytotoxic to either the neuro-2A and HCT-116 cell lines and we speculate that the *cis* configuration in the middle of the polyketide chain may affect binding of **2** to its molecular target. Smenothiazole A showed nanomolar levels against multiple cell lines and was previously isolated from a marine sponge; however, the authors indicate a likely cyanobacterial origin (Esposito et al., 2015). This is the first report of smenothiazole A from a bloom of *Trichodesmium*.

Overall, the networking procedure has identified new target molecules for isolation such as those in Cluster 1 (Figure 2). A comprehensive characterization of the chemical space within the bloom material is challenging as the metabolic composition of the sub-fractions we have generated are all nearly as complex as that of the sub-fraction from which **1–3** were isolated (Figure S2). The networking tool significantly improves the efficiency of our isolation and characterization workflow.

We did not identify anatoxins, saxitoxins or microcystins during the course of this analysis. This may be due to our focus on lipophilic metabolites, low abundance of these compounds in our samples, or a lack of informative MS/MS fragments. *Trichodesmium* blooms are complex events harboring a diverse array of microorganisms (Capone et al., 1997). Thus, the unequivocal identification of the producing organisms of the toxic metabolites described in this current work and other studies from environmental collections will ultimately require pure cultivation of producing organisms, the identification of biosynthetic gene clusters of toxic molecules, or the localization of metabolites to particular cell types (Simmons et al., 2008). In the original isolation and characterization of smenamides A and B, the authors suggest that a cyanobacterial symbiont is the true producer of the sponge-derived compounds (Teta et al., 2013). The present report supports this observation, as certain structural features such as the exomethylene vinyl chloride moiety are characteristic of cyanobacterial metabolism (Kan et al., 2000; Edwards et al., 2004; Nunnery et al., 2012). To the best of our knowledge, the *N*-methyl propanamide functionality in **3** has not previously been reported in a polyketide-peptide from cyanobacteria, and represents a biosynthetically intriguing unit because these organisms are not known to produce propionate. Conceivably, it may derive from an *S*-adenosylmethionine (SAM)-mediated methylation of an acetate precursor, the proposed first building block in the production of these smenamide-type natural products. This would be a similar biosynthetic transformation to that involved in producing the *t*-butyl group in apratoxin A which employs a combination of two SAM methyl transferases to incorporate these methyl groups (Grindberg et al., 2011; Skiba et al., 2017).

The identification of these neurotoxic metabolites (**1** and **3**) and the other more potent smenamides and smenothiazole A from a *Trichodesmium* bloom raises important questions as to their ecological role during these events. It will be important to characterize these bloom-associated metabolites in a longitudinal sense to evaluate their ongoing contribution to HABs.

## Author contributions

MB was responsible for the study design. PZ and PM were involved in sample collection, organism identification and sample storage. CV and MB did the structure characterization of secondary metabolites. CV and SC carried out cytotoxicity studies. EG, WG, and MB did the molecular networking procedure. MB wrote the manuscript with editing help from all co-authors.

### Conflict of interest statement

The authors declare that the research was conducted in the absence of any commercial or financial relationships that could be construed as a potential conflict of interest.

## Footnotes

^1^ProteoWizardSoftware Foundation; http://proteowizard.sourceforge.net/tools.shtml
