# Supplementary material for: The Metabolome of a Cyanobacterial Bloom Visualized by MS/MS-Based Molecular Networking Reveals New Neurotoxic Smenamide Analogs (C, D, and E)
Source: Front Chem. 2018 Jul 26;6:316. doi: 10.3389/fchem.2018.00316 (PMC6071517; doi:10.3389/fchem.2018.00316)
Supplement: Supplementary file 1 [file Table_1.DOCX]

Supporting Information: The complex metabolome of a cyanobacteria bloom visualized by MS/MS molecular networking

Christopher W. Via, Evgenia Glukhov, Samuel Costa, Paul V. Zimba, Peter D. R. Moeller, William H. Gerwick and Matthew J. Bertin

**Tables**

**Table S1.** NMR data for smenamide C (**1**) *E*-conformer (800 MHz for ^1^H NMR; 200 MHz for ^13^C NMR, DMSO).

**Table S2.** NMR data for smenamide C (**1**) *Z*-conformer (500 MHz for ^1^H NMR; 125 MHz for ^13^C NMR, CDCl_3_).

**Table S3.** NMR data for smenamide D (**2**) (CDCl_3_).

**Table S4.** NMR data for smenothiazole A.

**Table S5.** Molecular networking parameters.

**Figures**

**Figure S1.** Cytotoxicity of *Trichodesmium* bloom chemical fractions against Neuro-2A cells.

**Figure S2.** HPLC chromatogram of pre-fraction from which the **1-3** and smenothiazole were isolated.

**Figure S3.** ^1^H NMR of smenamide C (**1**) (800 MHz, DMSO).

**Figure S4.** ^13^C NMR of smenamide C (**1**) (200 MHz, DMSO).

**Figure S5.** HSQC of **1**.

**Figure S6.** HMBC of **1**.

**Figure S7.** COSY of **1**.

**Figure S8.** TOCSY of **1**.

**Figure S9.** NOESY of **1**.

**Figure S10.** ^1^H NMR of **1** (500 MHz, CDCl_3_).

**Figure S11.** HRESIMS of **1**.

**Figure S12.** MS/MS spectrum of **1**.

**Figure S13.** CD spectrum of **1** (80 μM in CH_3_OH).

**Figure S14.** ^1^H NMR of smenamide D (**2**) (800 MHz, CDCl_3_).

**Figure S15.** HSQC of **2**.

**Figure S16.** HMBC of **2**.

**Figure S17.** COSY of **2**.

**Figure S18.** TOCSY of **2**.

**Figure S19.** NOESY of **2**.

**Figure S20.** HRESIMS of **2**.

**Figure S21.** MS/MS spectrum of **2**.

**Figure S22.** ^1^H NMR of smenamide E (**3**) (800 MHz, DMSO).

**Figure S23.** ^13^C NMR of smendamide E (**3**) (200 MHz, DMSO).

**Figure S24.** HSQC of **3**.

**Figure S25.** HMBC of **3**.

**Figure S26.** COSY of **3**.

**Figure S27.** TOCSY of **3**.

**Figure S28.** NOESY of **3**.

**Figure S29.** HRESIMS of **3**.

**Figure S30.** MS/MS spectrum of **3**.

**Figure S31.** ^1^H NMR of smenothiazole A (800 MHz, DMSO).

**Figure S32.** ^13^C NMR of smenothiazole A (200 MHz, DMSO).

**Figure S33.** HRESIMS of smenothiazole A.

**Figure S34.** MS/MS spectrum of smenothiazole A.

**Figure S35.** CD spectrum of smenothiazole A (CH_3_OH).

**Figure S36.** MS/MS of smenamide A/B detected in *Trichodesmium* chemical fractions.

**Figure S37.** Dose-response curves of **1-3** against HCT-116 and neuro-2A cells.

Table S1. NMR data for smenamide C (**1**) *E*-conformer (800 MHz for ^1^H NMR; 200 MHz for ^13^C NMR, DMSO)

position *δ*_C_, type *δ*_H_ (*J* in Hz)

1 169.3, qC

2 93.7, CH 5.28, s

3 180.3, qC

4 57.6, CH 4.75, t (5.3)

5a 38.6, CH_2_ 1.72, m

5b 1.59, m

6 24.2, CH 1.66, m

7 24.0, CH_3_ 0.85, d (6.6)

8 23.3, CH_3_ 0.87, d (6.6)

9 59.7, CH_3_ 3.87, s

10 170.6, qC

11 131.7, qC

12 13.9, CH_3_ 1.77, s

13 141.9, CH 5.59, m

14 32.1, CH 2.45, m

15 20.5, CH_3_ 0.94, d (6.6)

16a 35.0, CH_2_ 1.49, m

16b 1.35, m

17a 32.2, CH_2_ 2.22, m

17b 2.10, ovlp^a^

18 142.6, qC

19 112.7, CH 6.07, s

20a 27.4, CH_2_ 2.12, m

20b 2.07, ovlp

21 25.7, CH_2_ 1.64, m

22 50.0, CH_2_ 3.27, m

23 32.8. CH_3_ 2.79, s

24 169.6, qC

25 21.6, CH_3_ 1.98, s

^a^overlapping signals

Table S2. NMR data for smenamide C (**1**) *Z*-conformer (500 MHz for ^1^H NMR; 125 MHz for ^13^C NMR, CDCl_3_)

position *δ*_C_, type *δ*_H_ (*J* in Hz)

1 169.2, qC

2 93.1, CH 4.97, s

3 180.0, qC

4 57.8, CH 4.80, m

5a 39.0, CH_2_ 1.79, m

5b 1.69, m

6 24.3, CH 1.73, m

7 23.6, CH_3_ 0.93, d (6.4)

8 22.6, CH_3_ 0.91, d (6.4)

9 58.5, CH_3_ 3.85, s

10 170.5, qC

11 131.8, qC

12 13.7, CH_3_ 1.88, s

13 142.7, CH 5.70, m

14 32.3, CH 2.49, m

15 20.5, CH_3_ 1.00, d (6.6)

16a 35.0, CH_2_ 1.53, m

16b 1.43, m

17a 32.2, CH_2_ 2.33, m

17b 2.10, ovlp^a^

18 142.6, qC

19 113.2, CH 5.94, s

20a 27.3, CH_2_ 2.23, m

20b 2.16, ovlp

21 25.7, CH_2_ 1.64, m

22 47.1, CH_2_ 3.40, m

23 35.9. CH_3_ 3.00, s

24 170.6, qC

25 21.8, CH_3_ 2.09, s

^a^overlapping signals

Table S3. NMR data for smenamide D (**2**) (CDCl_3_)^a^

*Z*-conformer *E*-conformer

position *δ*_C_, type *δ*_H_ (*J* in Hz) *δ*_C_, type *δ*_H_ (*J* in Hz)

1 168.6, qC 168.6, qC

2 93.2, CH 5.02, s 93.2, CH 5.02, s

3 180.7, qC 180.7, qC

4 58.0, CH 4.71, m 58.0, CH 4.71, m

5a 38.9, CH_2_ 1.87, m 38.9, CH_2_ 1.87, m

5b 1.79, m 1.79, m

6 24.2, CH 1.76, m 24.2, CH 1.76, m

7 23.9, CH_3_ 0.93, d (6.4) 23.9, CH_3_ 0.93, d (6.4)

8 22.8, CH_3_ 0.90, d (6.4) 22.8, CH_3_ 0.90, d (6.4)

9 58.6, CH_3_ 3.87, s 58.6, CH_3_ 3.87, s

10 169.5, qC 169.5, qC

11 132.6, qC 132.6, qC

12 20.1, CH_3_ 1.93, s 20.1, CH_3_ 1.93, s

13 135.9, CH 5.17, d (10.4) 135.9, CH 5.17, d (10.4)

14 34.2, CH 2.16, m 34.2, CH 2.16, m

15 21.2, CH_3_ 0.99, d (6.7) 21.2, CH_3_ 0.99, d (6.7)

16a 35.0, CH_2_ 1.37, m 35.0, CH_2_ 1.37, m

16b 1.30, m 1.30, m

17a 32.5, CH_2_ 2.08, m 32.5, CH_2_ 2.08, m

17b 1.89, ovlp^b^ 1.89, ovlp

18 141.3, qC 142.0, qC

19 113.0, CH 5.80, s 112.4, CH 5.74, s

20a 27.5, CH_2_ 2.16, m 27.4, CH_2_ 2.16, m

20b 2.12, ovlp 2.12, ovlp

21 24.6, CH_2_ 1.61, m 25.6, CH_2_ 1.68, m

22 47.2, CH_2_ 3.37, t (7.5) 50.5, CH_2_ 3.26, t (7.5)

23 36.1 CH_3_ 2.99, s 33.2, CH_3_ 2.92, s

24 170.7, qC 170.6, qC

25 21.9, CH_3_ 2.08, s 21.2, CH_3_ 2.10, s

^a^800 MHz for ^1^H NMR, ^13^C NMR from HSQC and HMBC spectra

^b^overlapping signals

Table S4. NMR data for smenothiazole A

residue position *δ*_C_, type *δ*_H_ (*J* in Hz)

Thiazole 1 142.5, CH 7.72, d (3.3)

2 120.2, CH 7.60, d (3.3)

Proline 3 172.7, qC

4 58.5, CH 5.37, dd (8.1, 2.7)

5a 31.9, CH_2_ 2.19, m

5b 2.14, m

6 24.5, CH_2_ 2.00, m

7a 47.5, CH_2_ 3.90, m

7b 3.77, m

Valine 8 171.4, qC

9 56.7, CH 4.40, t (8.3)

10 30.4, CH 2.11, m

11 19.6, CH_3_ 0.91, d (6.7)

12 19.2, CH_3_ 0.90, d (6.7)

NH 7.77, d (8.3)

PKS 13 168.8, qC

14 132.8, qC

15 13.4, CH_3_ 1.73, s

16 130.7, CH 6.15, m

17 29.2, CH_2_ 2.90, t (6.5)

18 140.5, qC

19 115.0, CH 6.30, s

20 40.5, CH_2_ 3.44, d (2.9)

21 138.5, qC

22/26 129.2, CH 7.20, d (7.6)

23/25 129.0, CH 7.32, t (7.6)

24 127.0, CH 7.24, t (7.6)

Table S5. Molecular networking parameters

Parameter Value

| PAIRS_MIN_COSINE | 0.6 |
| --- | --- |
| ANALOG_SEARCH | 1 |
| tolerance.PM_tolerance | 2.0 |
| tolerance.Ion_tolerance | 0.9 |
| MIN_MATCHED_PEAKS | 3 |
| TOPK | 10 |
| CLUSTER_MIN_SIZE | 1 |
| MAXIMUM_COMPONENT_SIZE | 100 |
| MIN_PEAK_INT | 0.0 |
| FILTER_STDDEV_PEAK_INT | 0.0 |
| RUN_MSCLUSTER | on |
| FILTER_PRECURSOR_WINDOW | 1 |
| FILTER_LIBRARY | 1 |
| WINDOW_FILTER | 1 |
| SCORE_THRESHOLD | 0.6 |
| MIN_MATCHED_PEAKS_SEARCH | 3 |
| MAX_SHIFT_MASS | 100.0 |

**
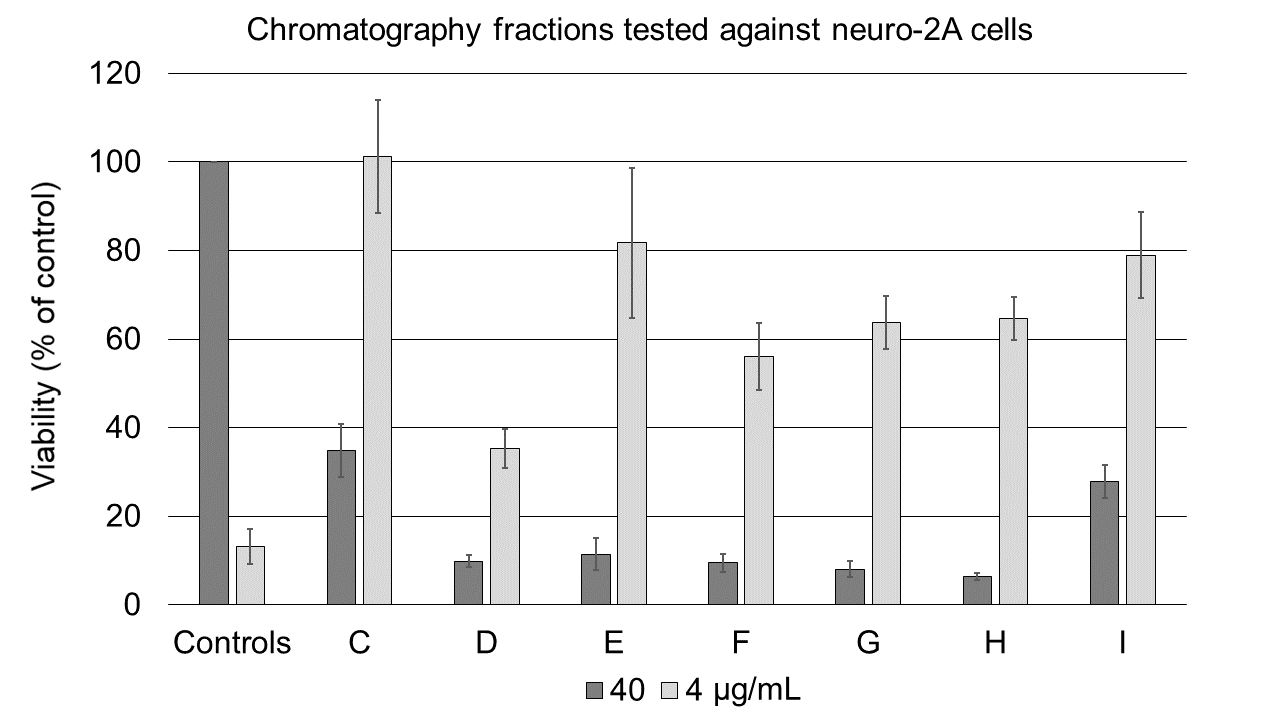
**

**Figure S1.** Cytotoxicity of *Trichodesmium* bloom chromatography fractions against neuro-2A cells.

**
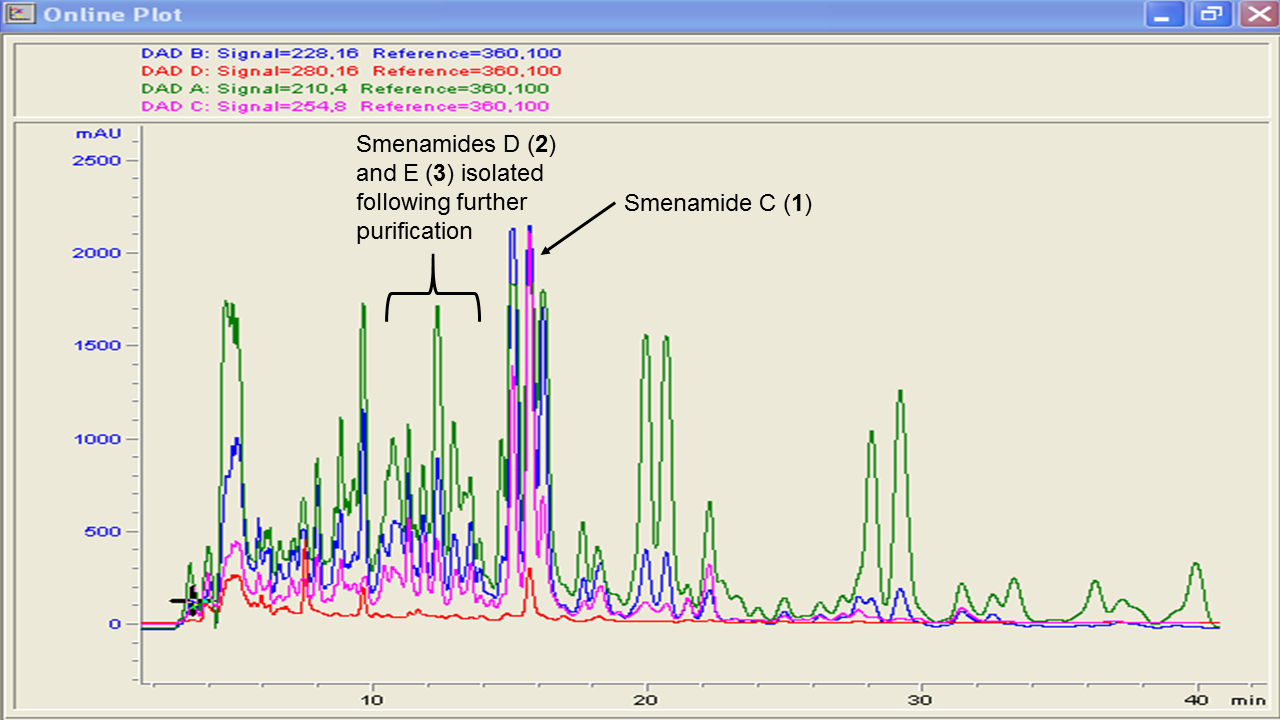
**

**Figure S2.** HPLC chromatogram of pre-fraction from which the **1-3** and smenothiazole were isolated.

**
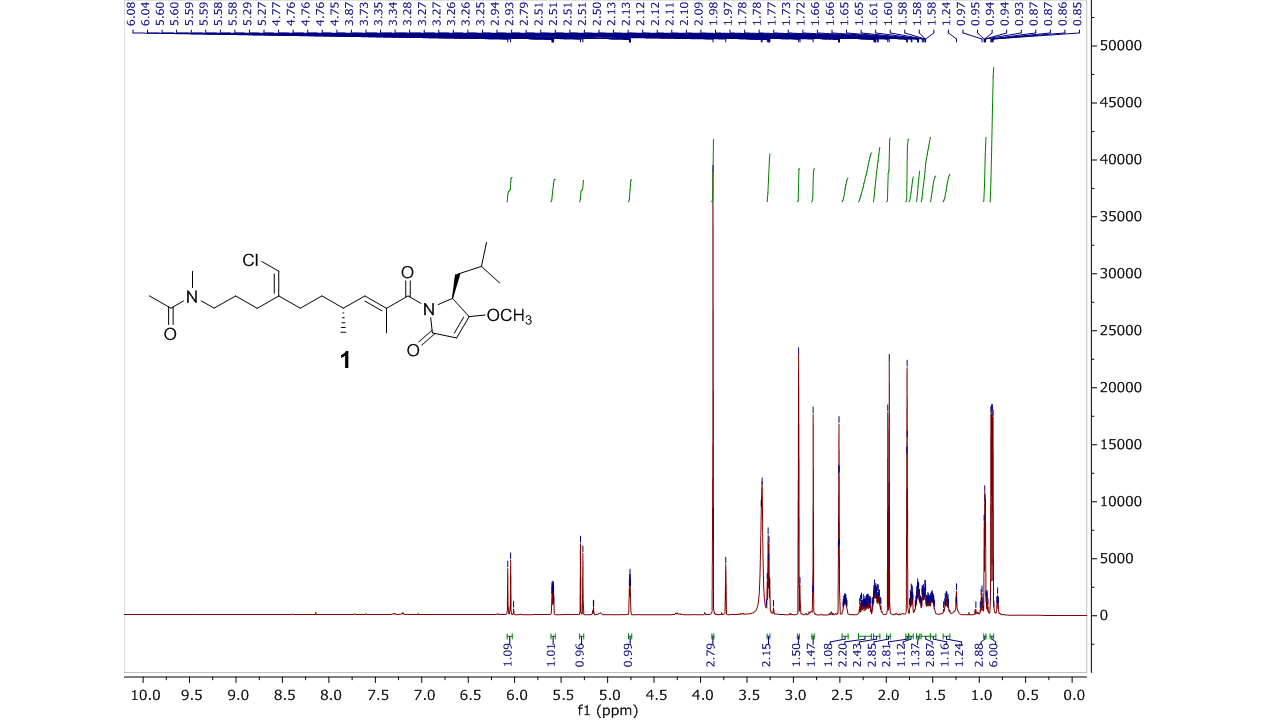
**

**Figure S3.** ^1^H NMR of smenamide C (**1**) (800 MHz, DMSO).

**
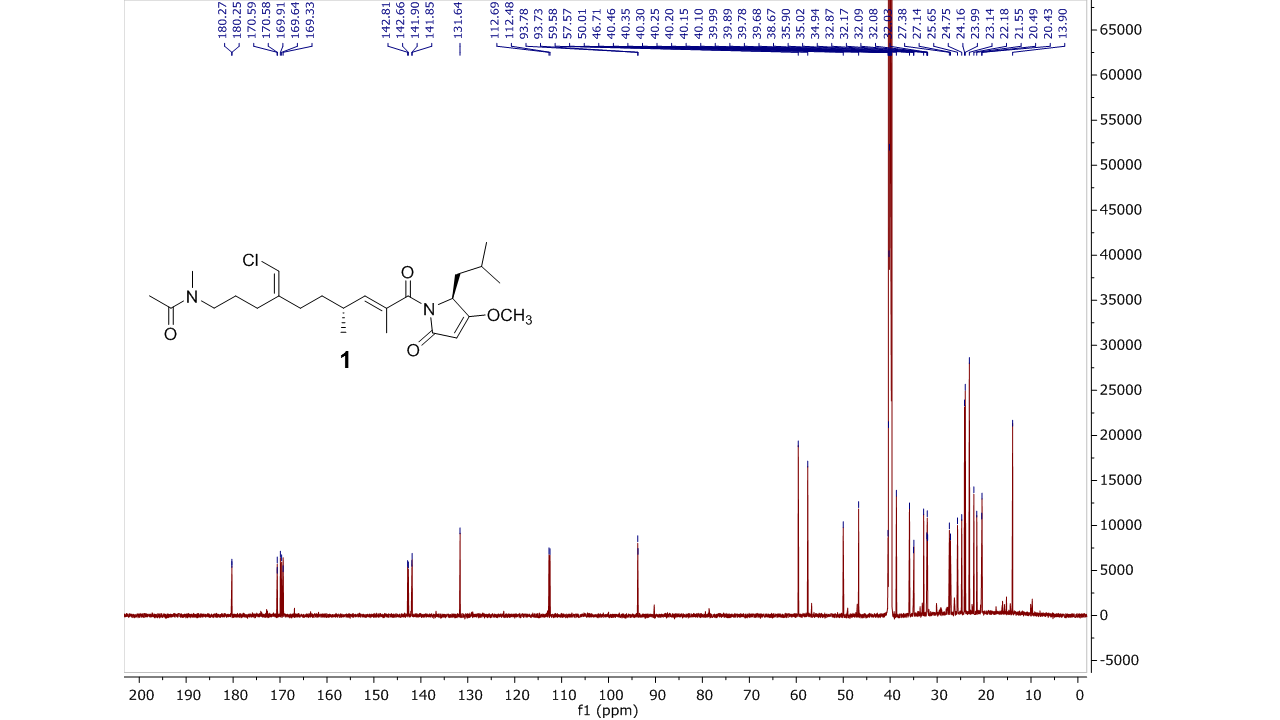
**

**Figure S4.** ^13^C NMR of smendamide C (**1**) (200 MHz, DMSO).

**
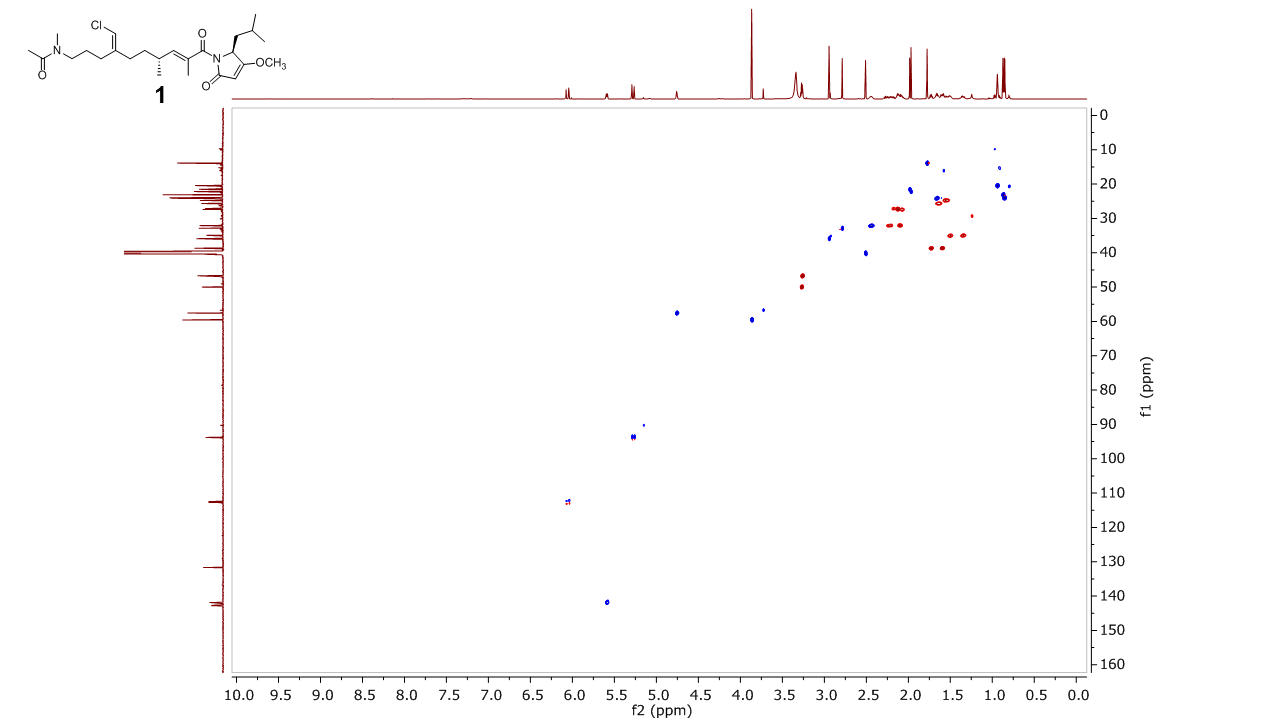
**

**Figure S5.** HSQC of **1**.

**
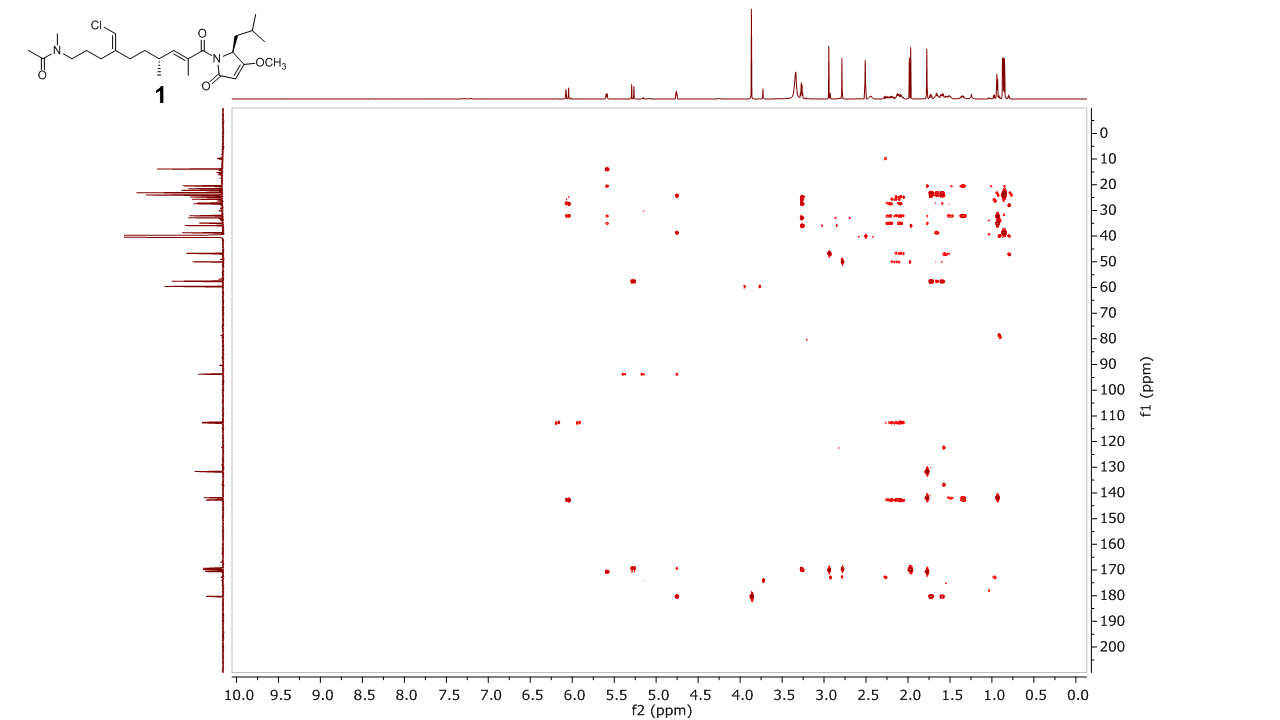
**

**Figure S6.** HMBC of **1**.

**
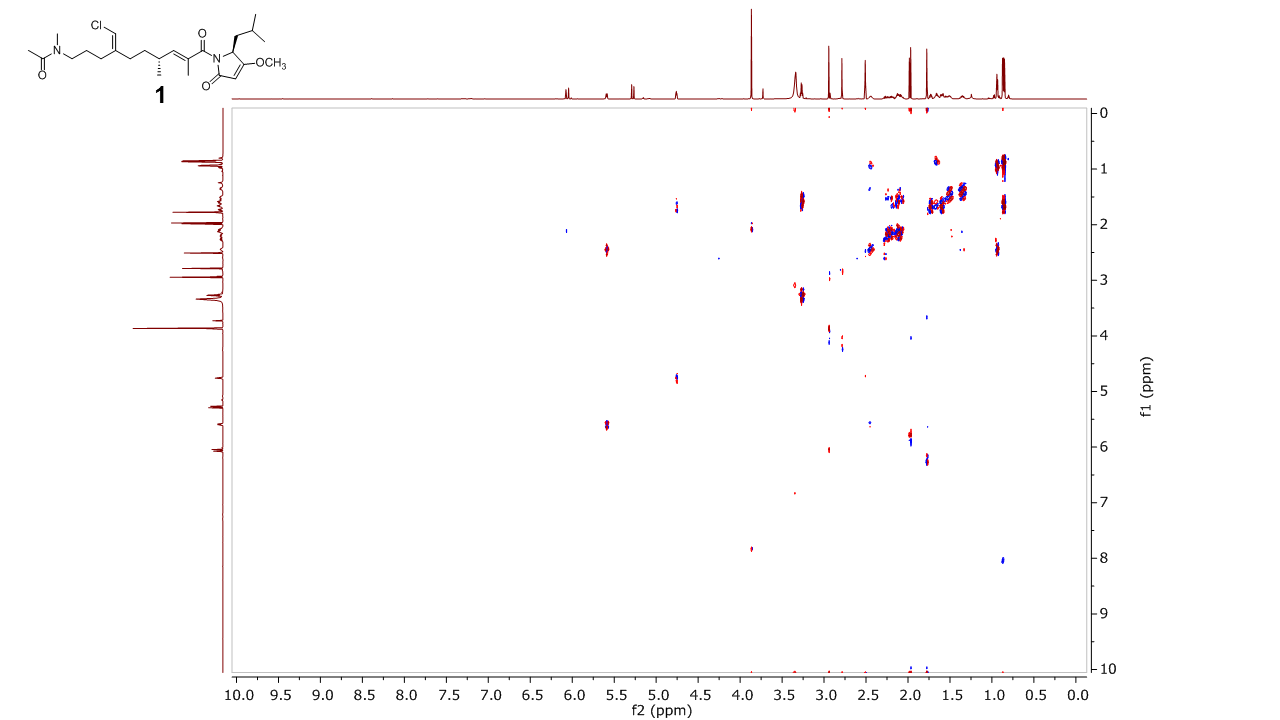
**

**Figure S7.** COSY of **1**.

**
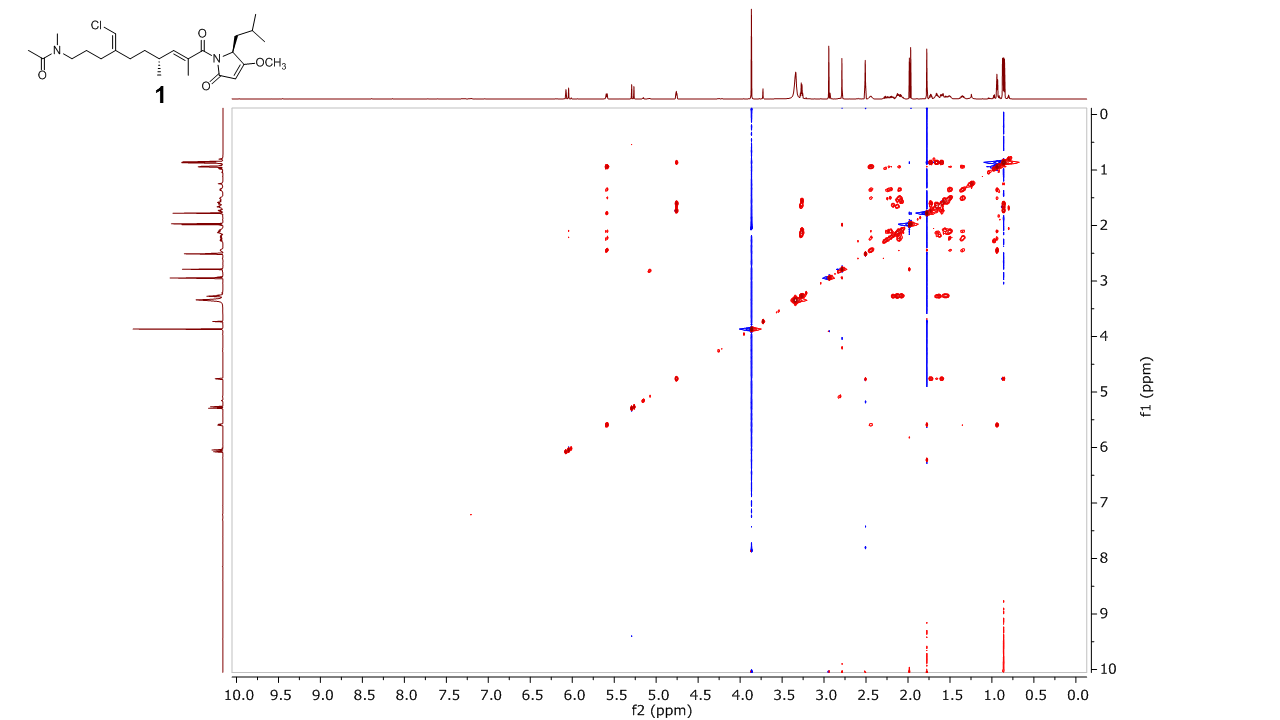
**

**Figure S8.** TOCSY of **1**.

**
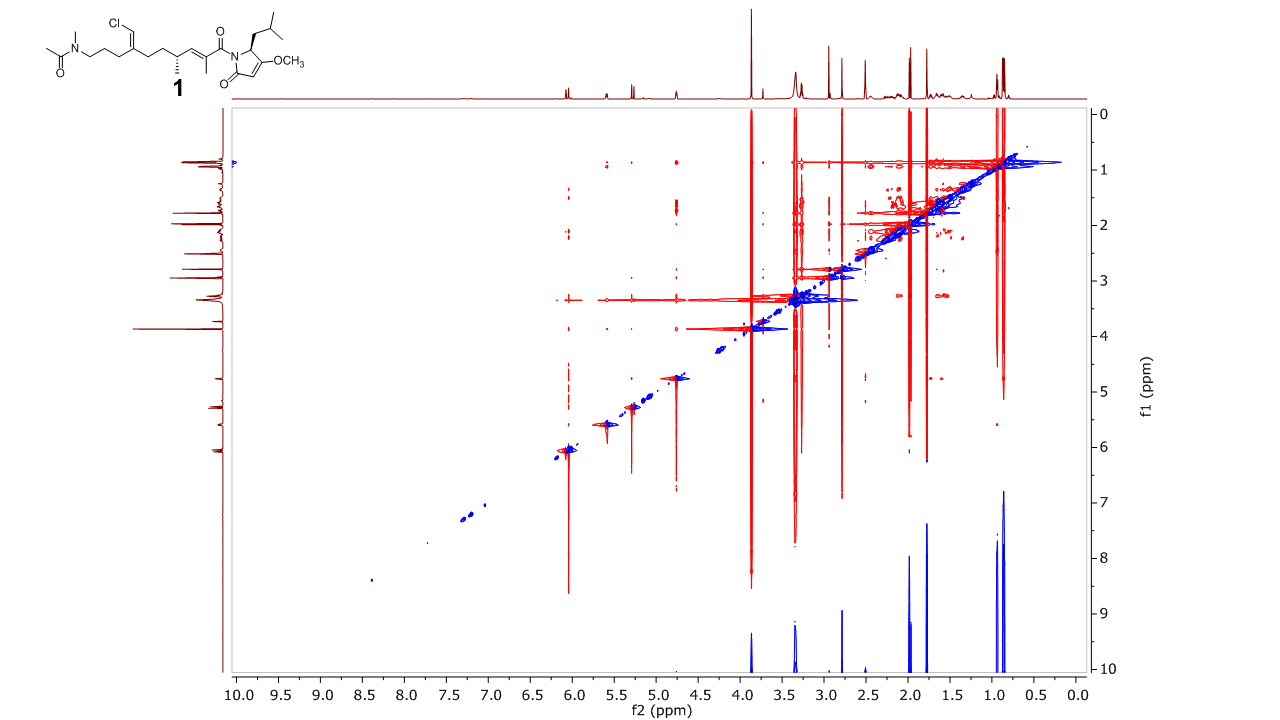
**

**Figure S9.** NOESY of **1**.

**
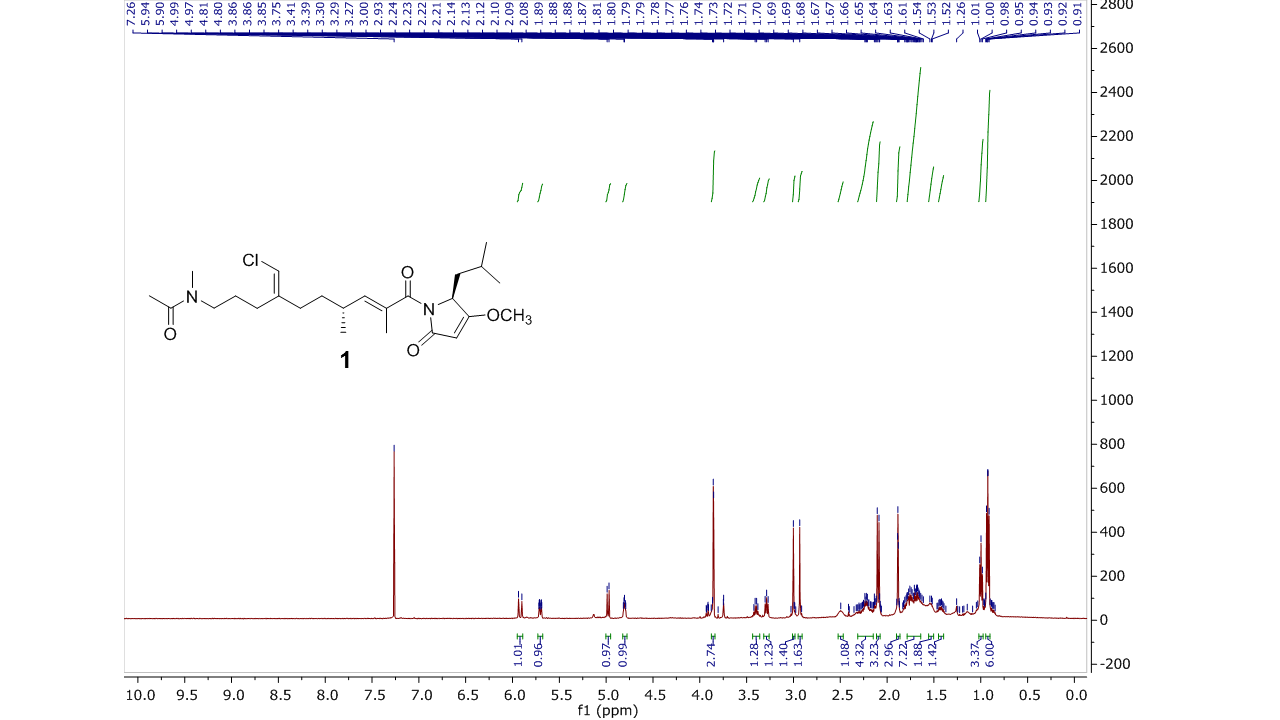
**

**Figure S10.** ^1^H NMR of **1** (500 MHz, CDCl_3_).

**
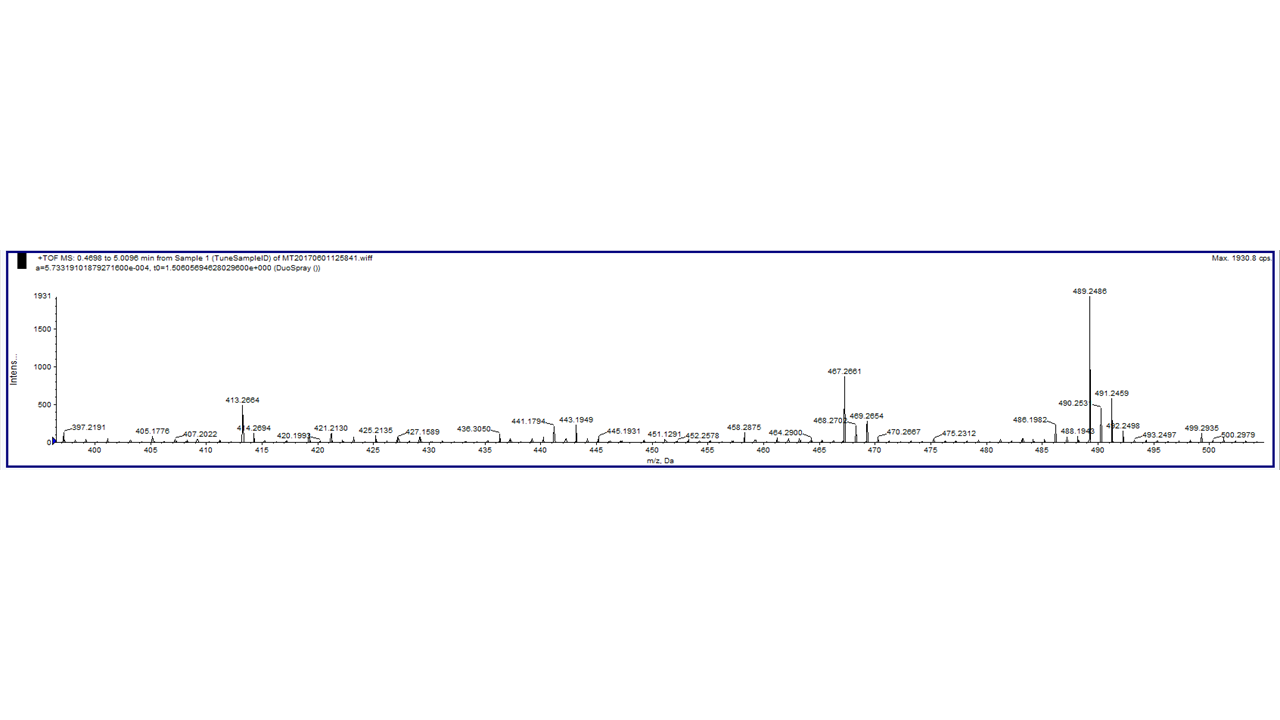
**

**Figure S11.** HRESIMS of **1**.

**
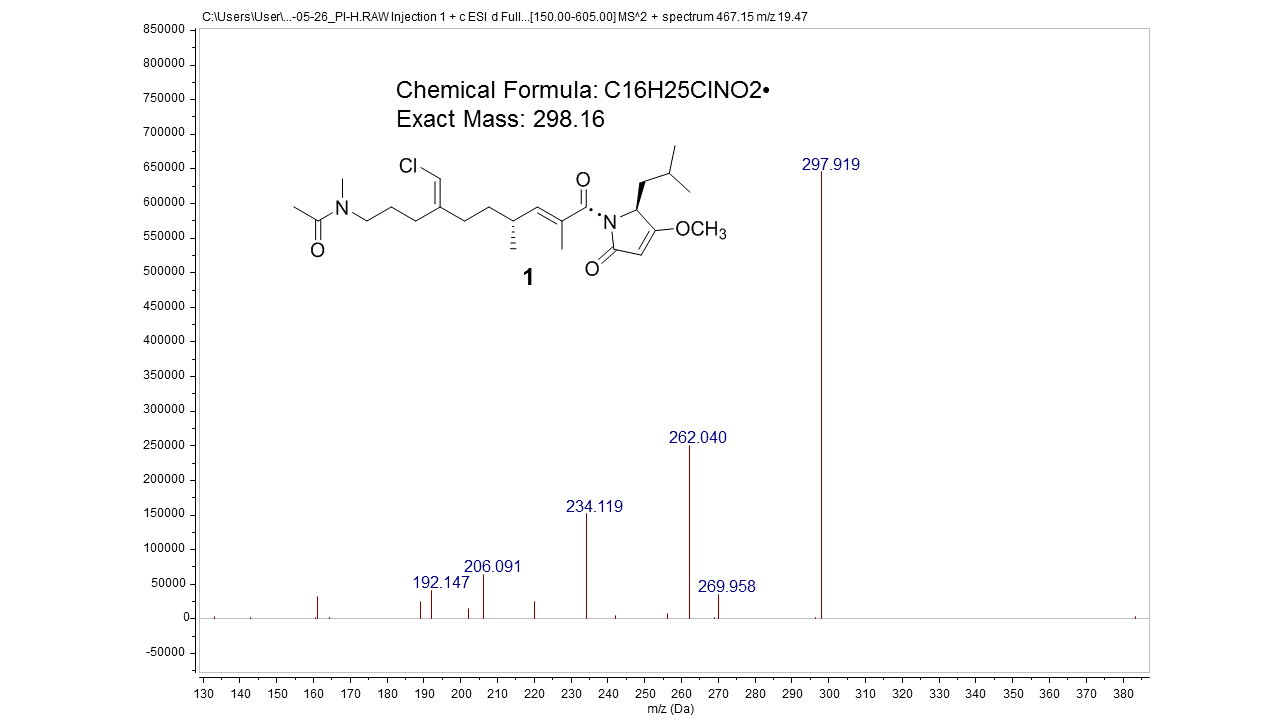
**

**Figure S12.** MS/MS spectrum of **1**.

**
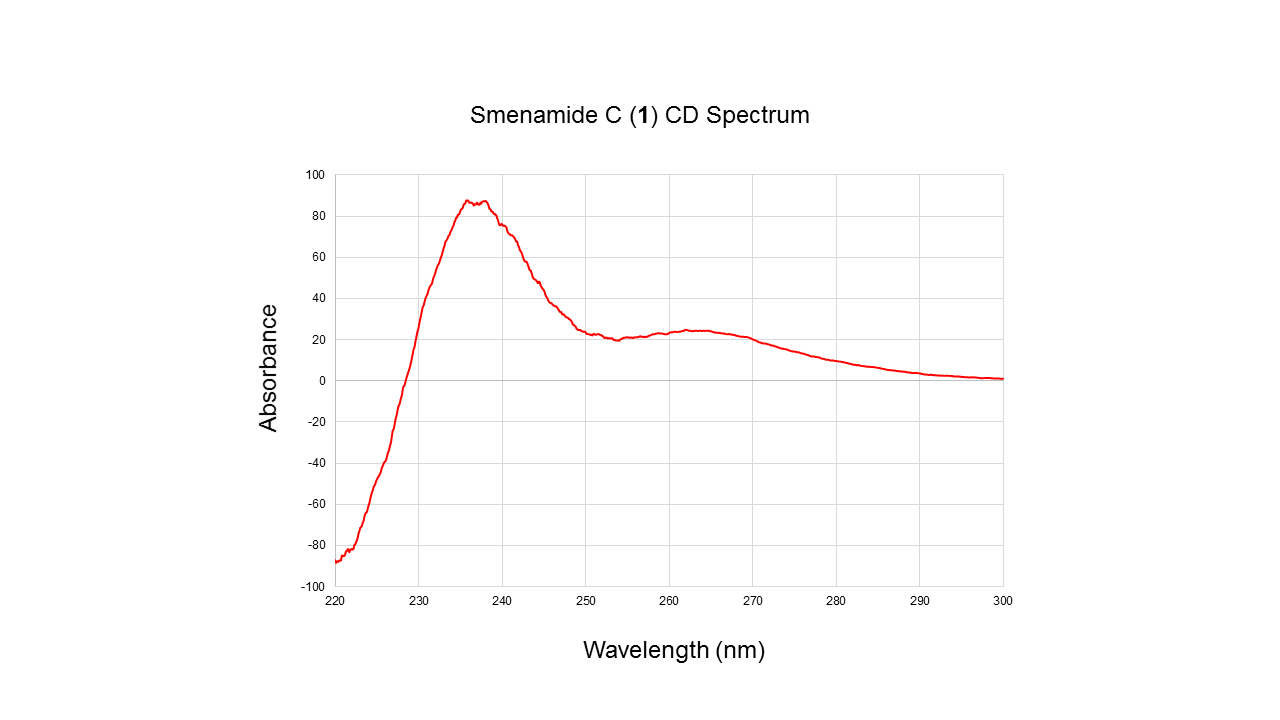
**

**Figure S13.** CD spectrum of **1** (80 μM in CH_3_OH).

**
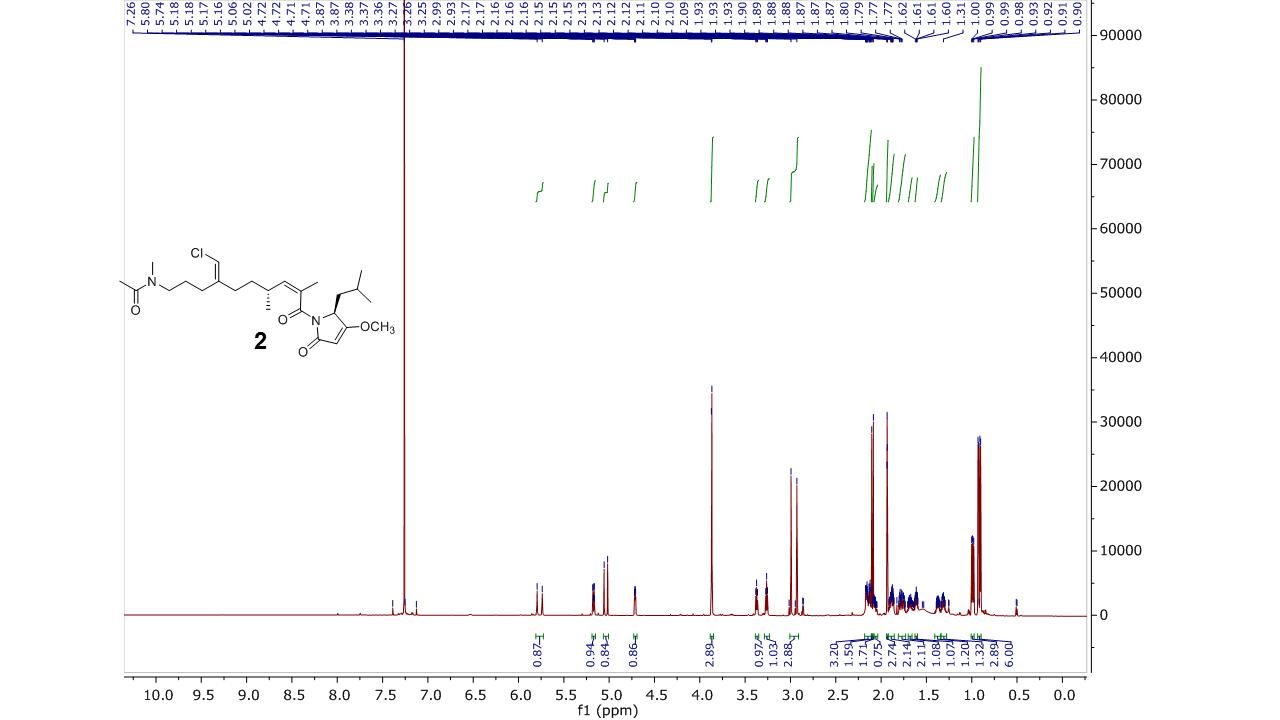
**

**Figure S14.** ^1^H NMR of smenamide D (**2**) (800 MHz, CDCl_3_).

**
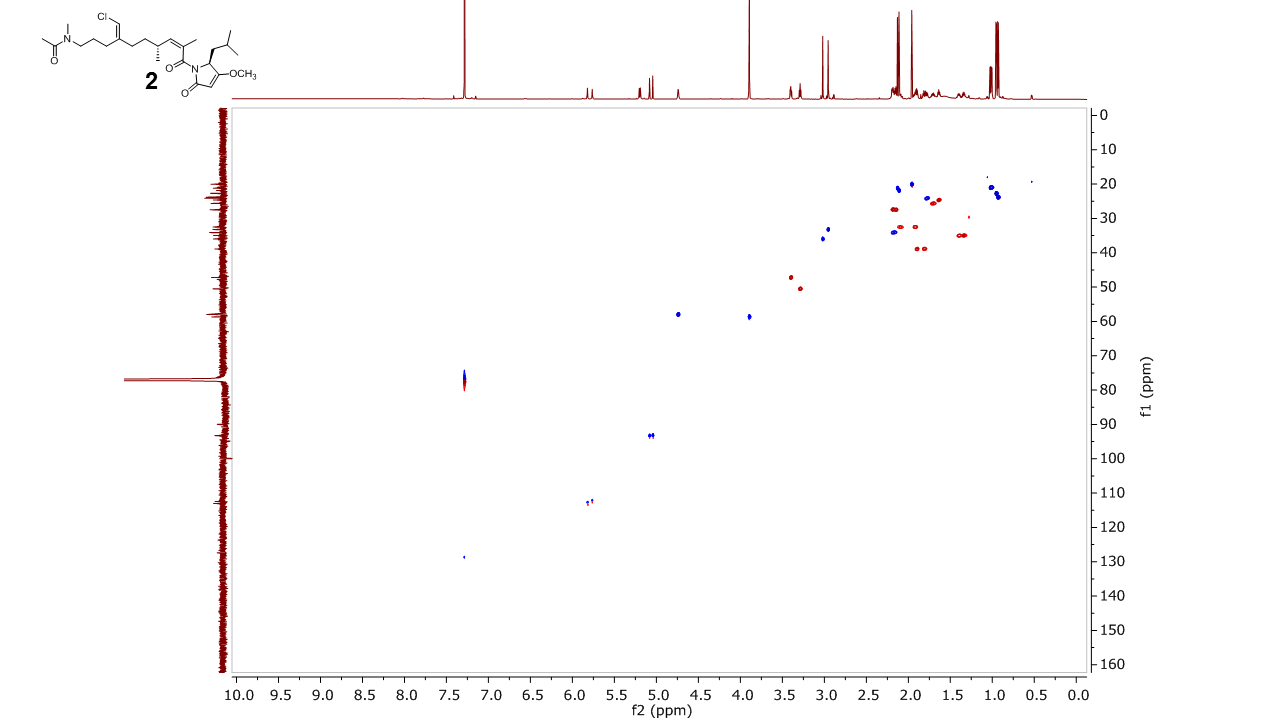
**

**Figure S15.** HSQC of **2**.

**
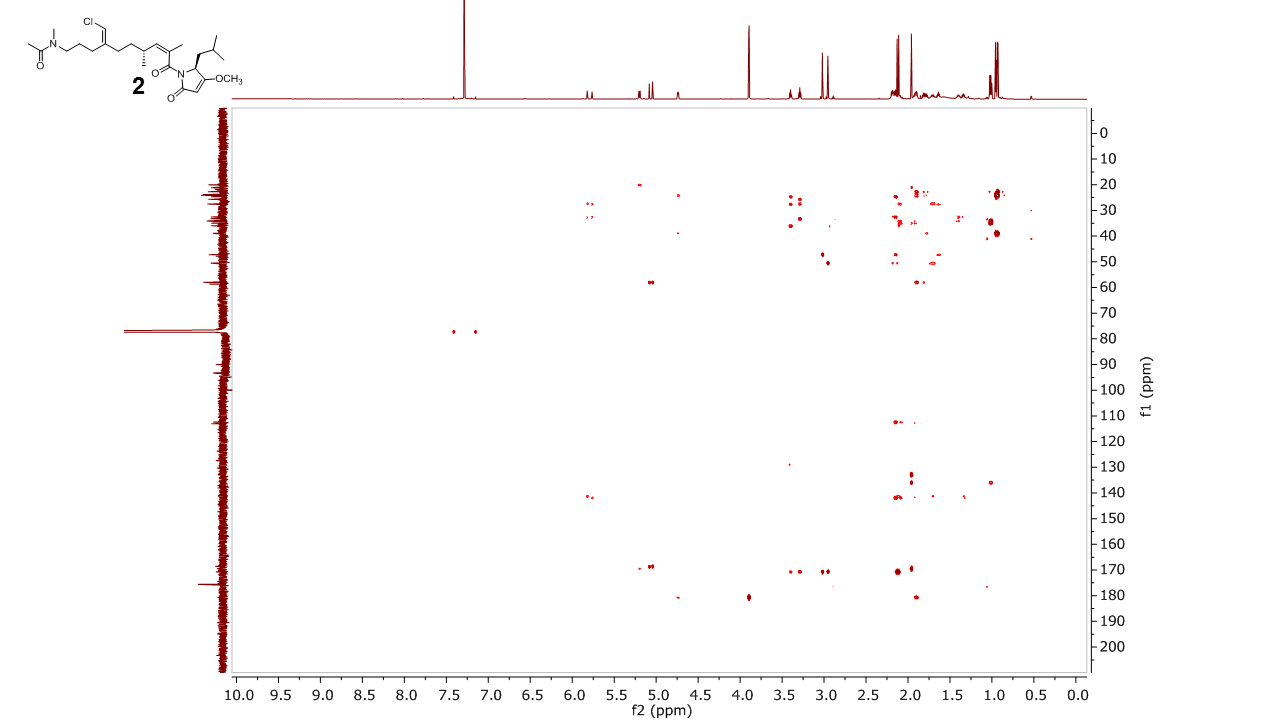
**

**Figure S16.** HMBC of **2**.

**
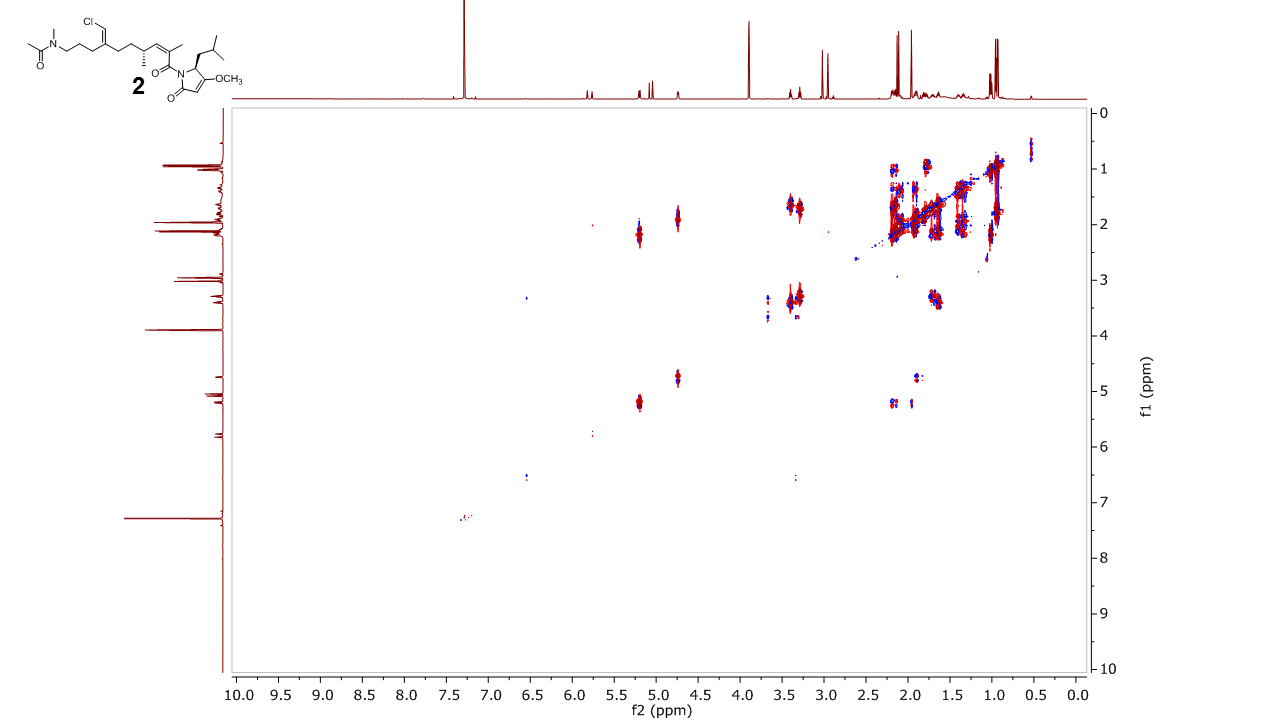
**

**Figure S17.** COSY of **2**.

**
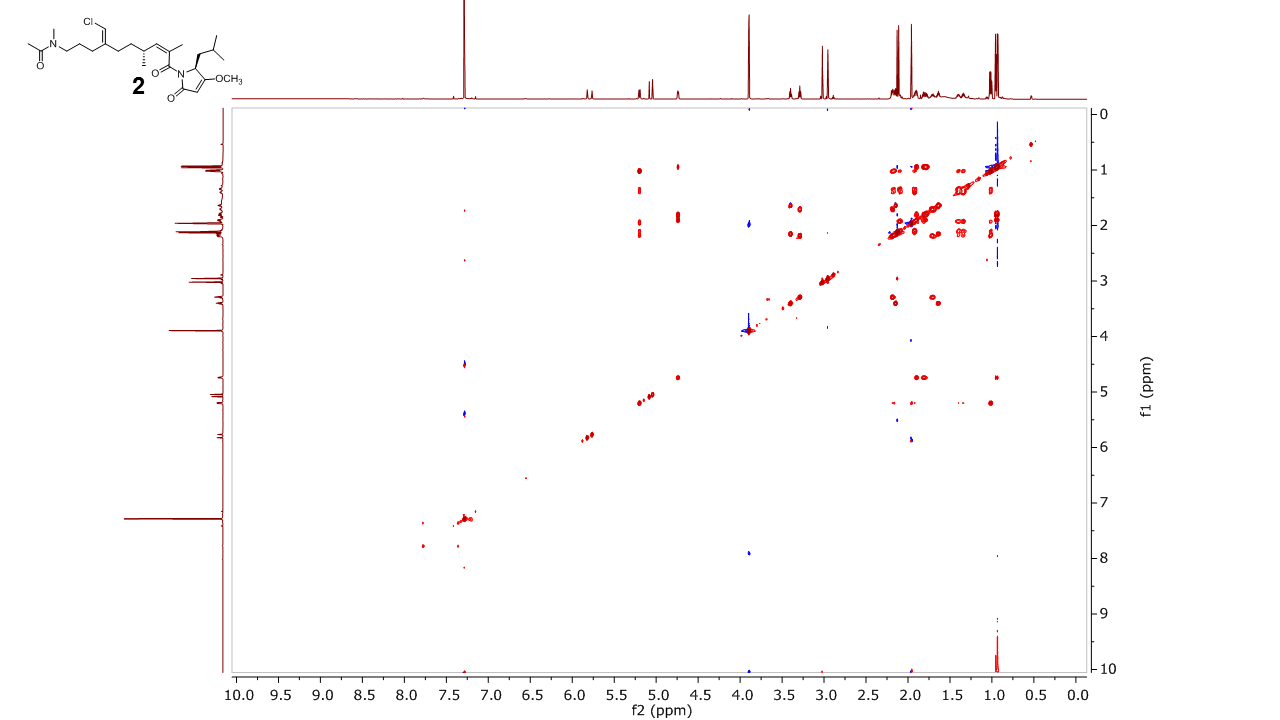
**

**Figure S18.** TOCSY of **2**.

**
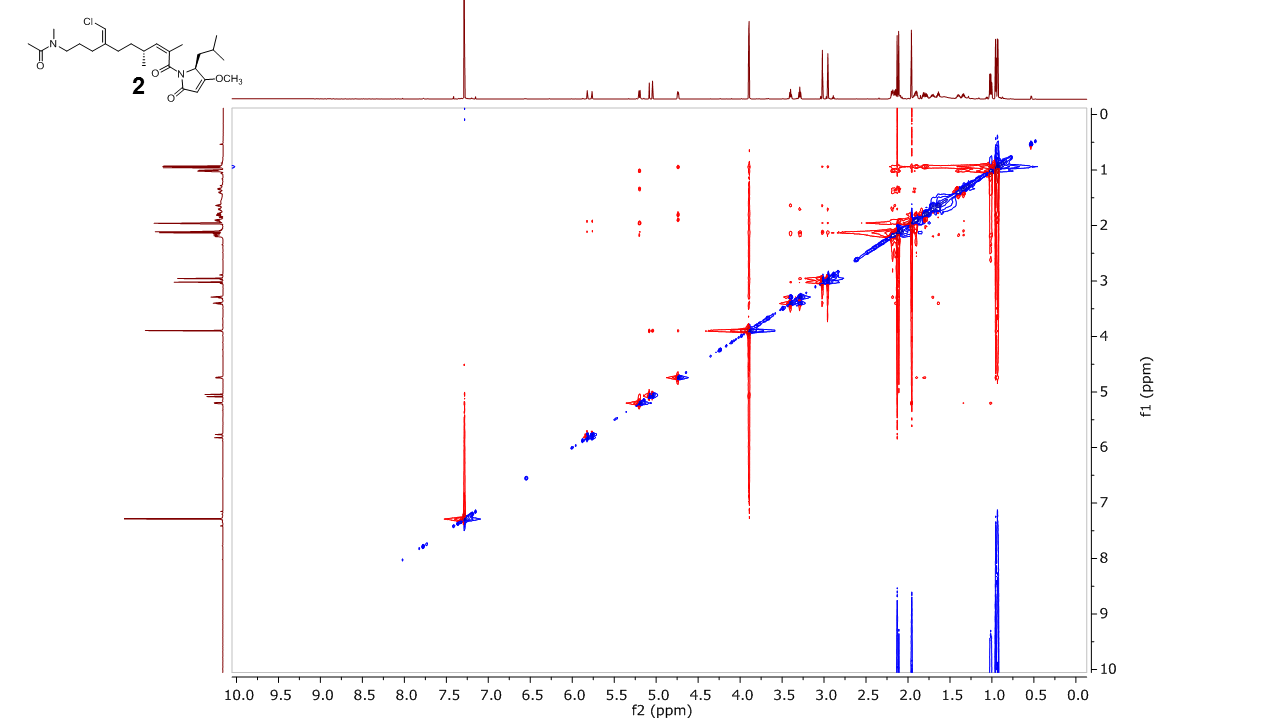
**

**Figure S19.** NOESY of **2**.

**
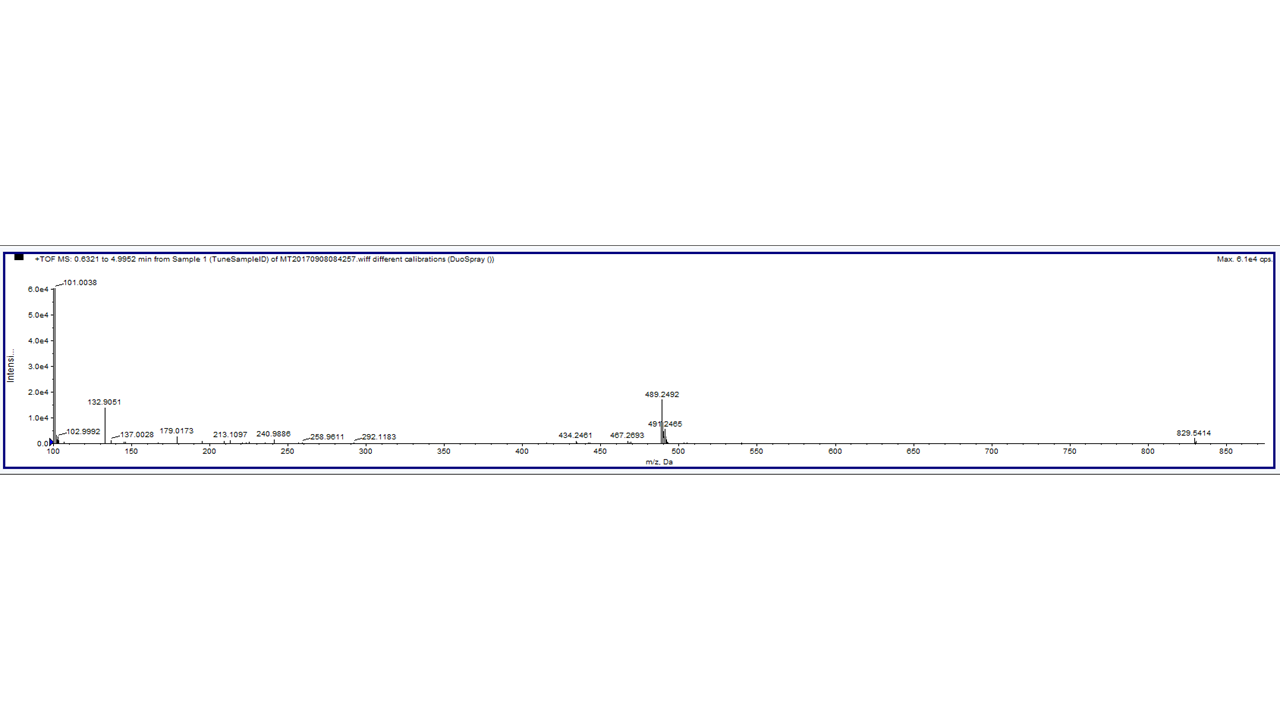
**

**Figure S20.** HRESIMS of **2**.

**
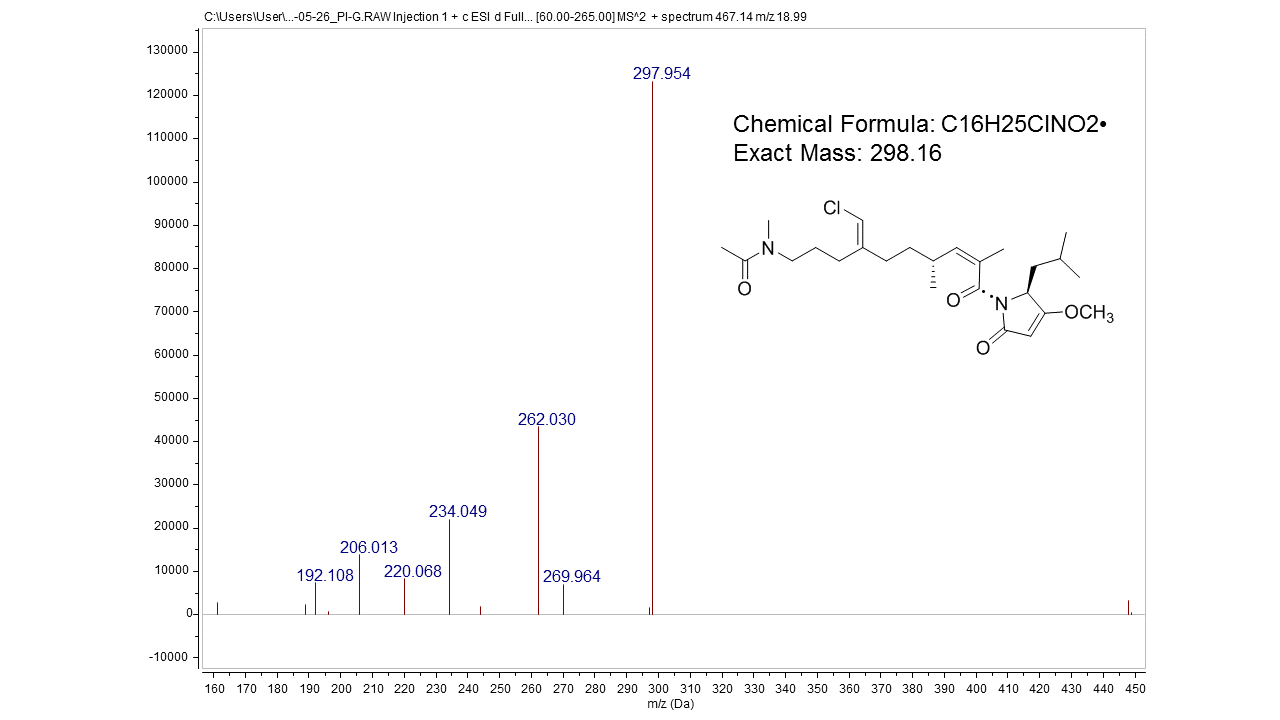
**

**Figure S21.** MS/MS spectrum of **2**.

**
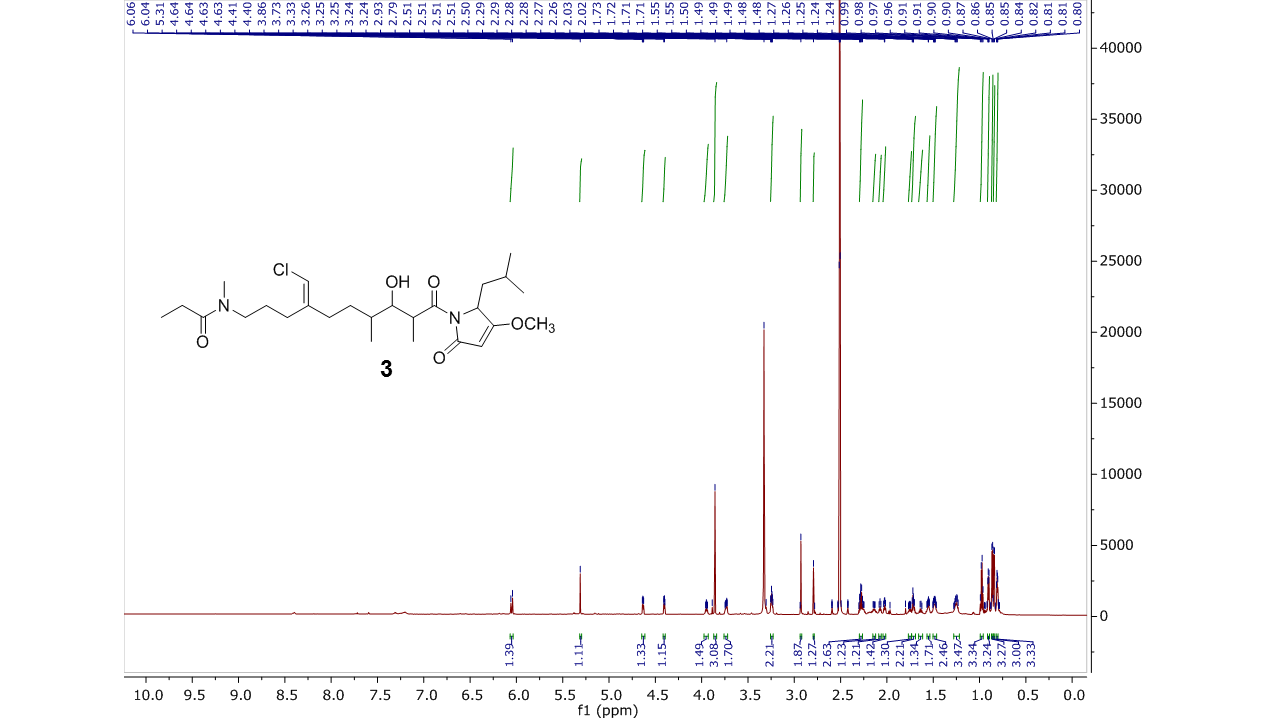
**

**Figure S22.** ^1^H NMR of smenamide E (**3**) (800 MHz, DMSO).

**
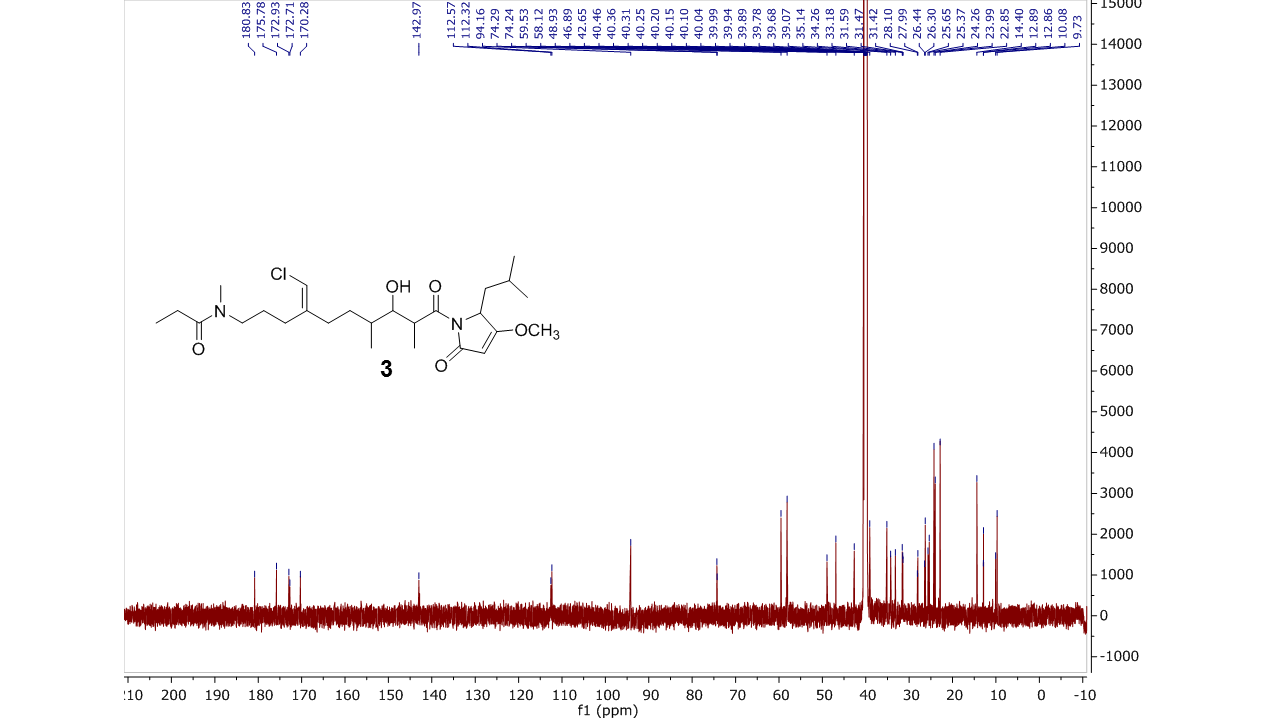
**

**Figure S23.** ^13^C NMR of smendamide E (**3**) (200 MHz, DMSO).

**
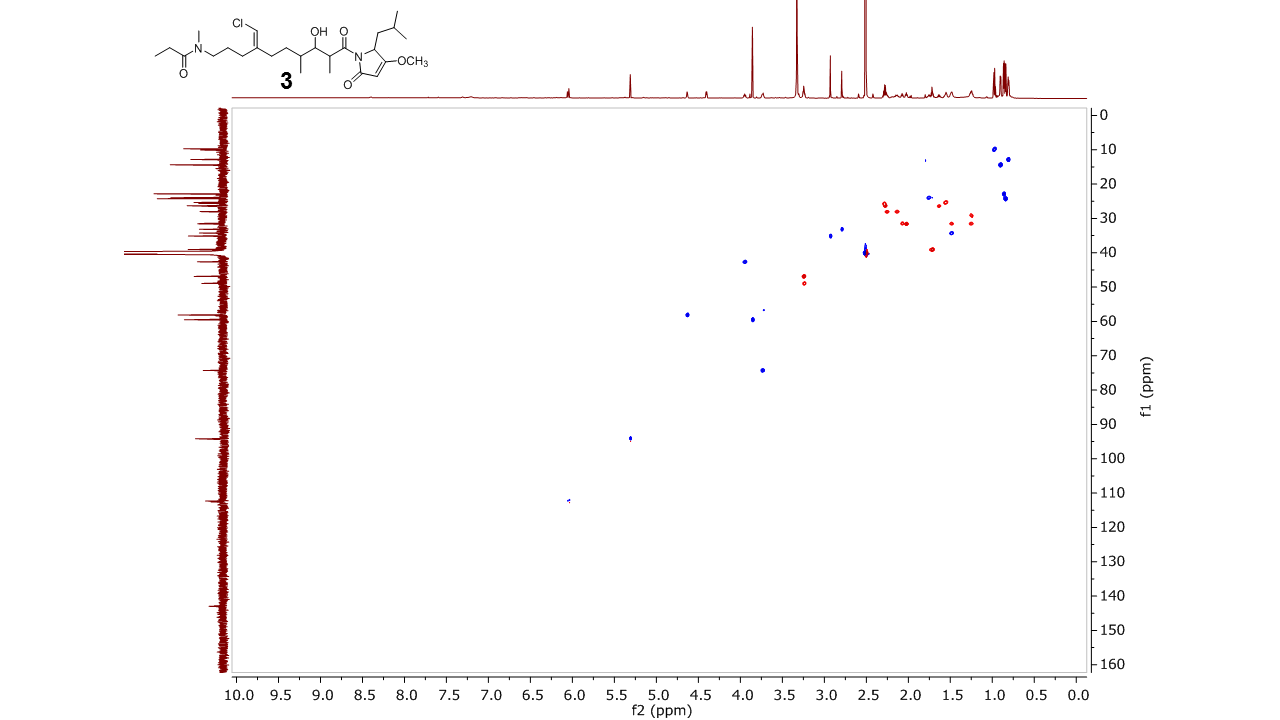
**

**Figure S24.** HSQC of **3**.

**
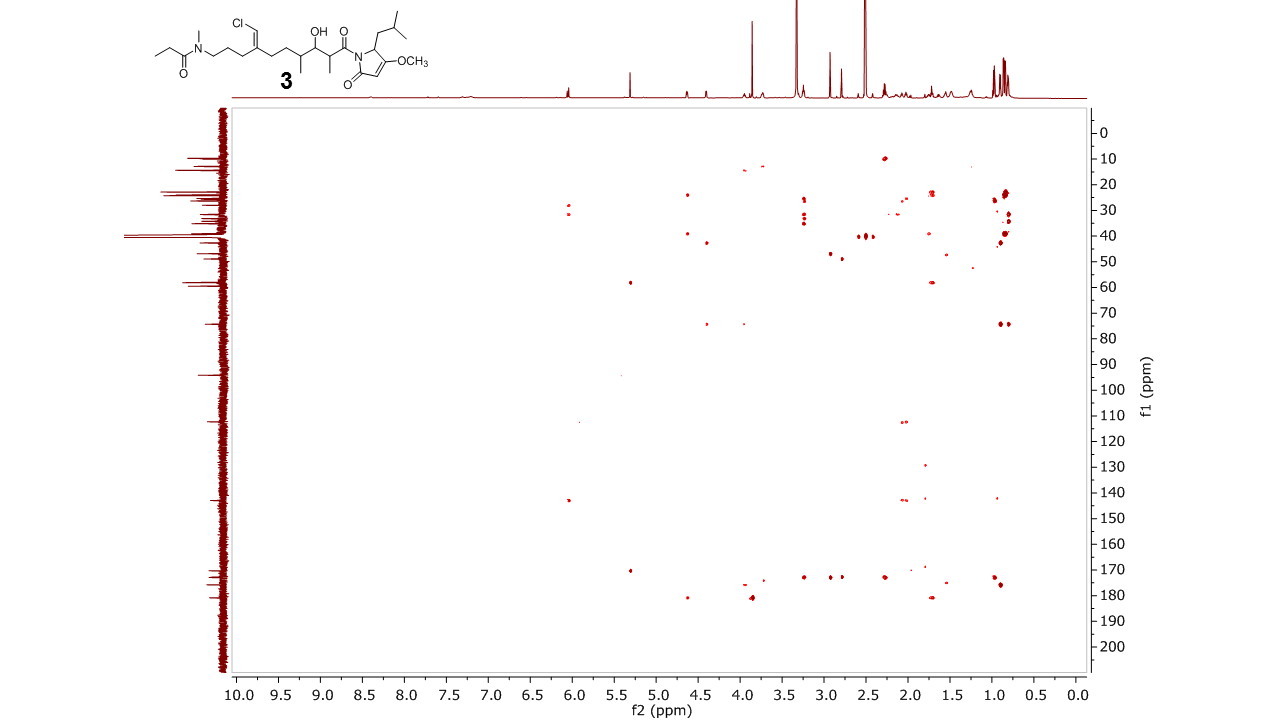
**

**Figure S25.** HMBC of **3**.

**
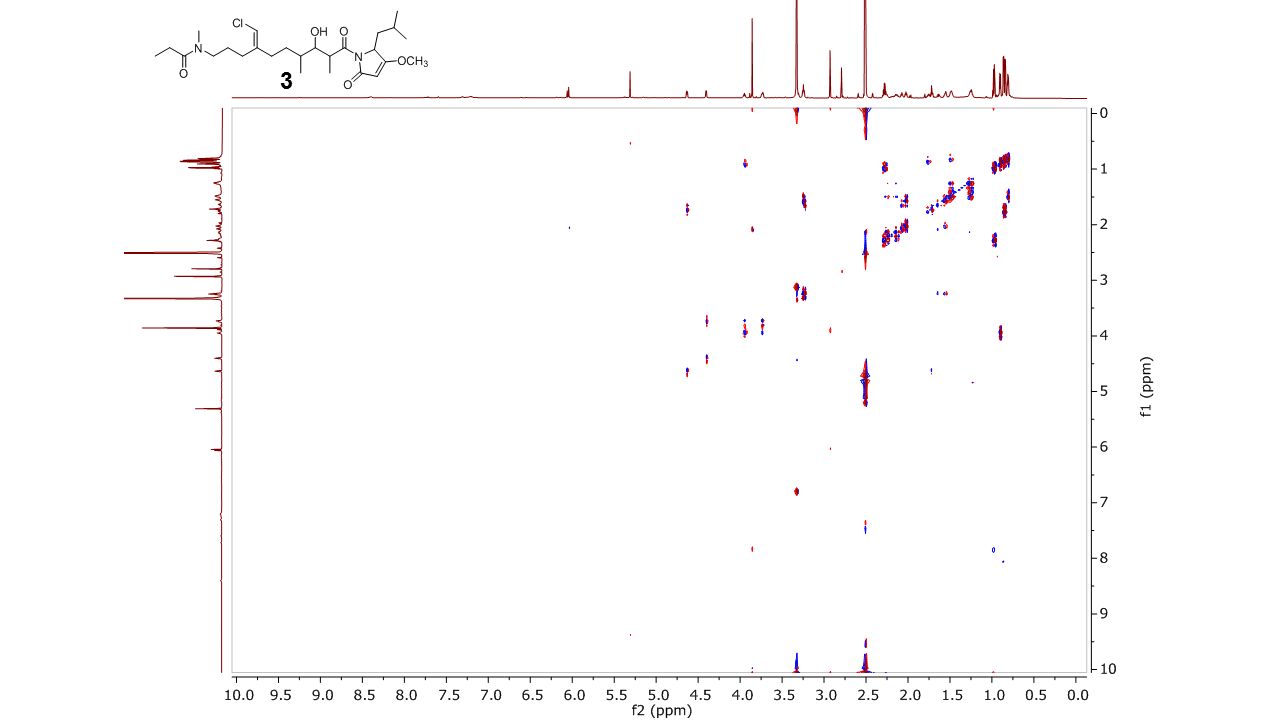
**

**Figure S26.** COSY of **3**.

**
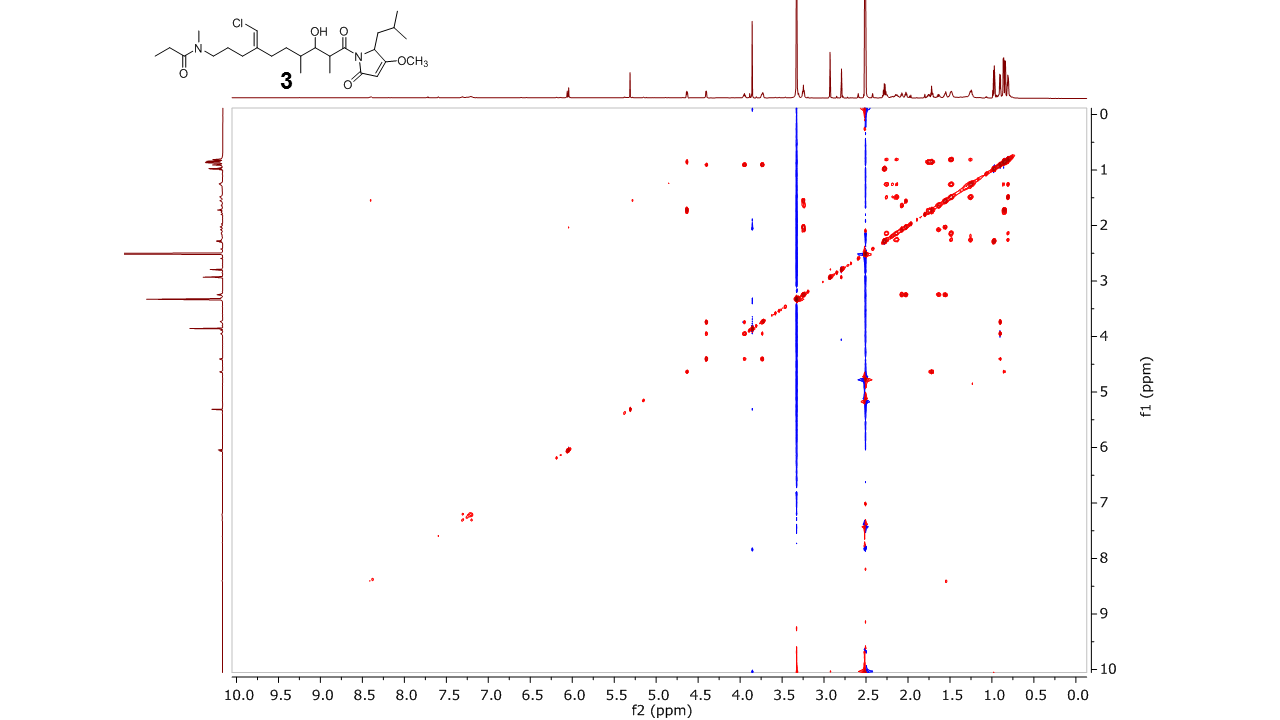
**

**Figure S27.** TOCSY of **3**.

**
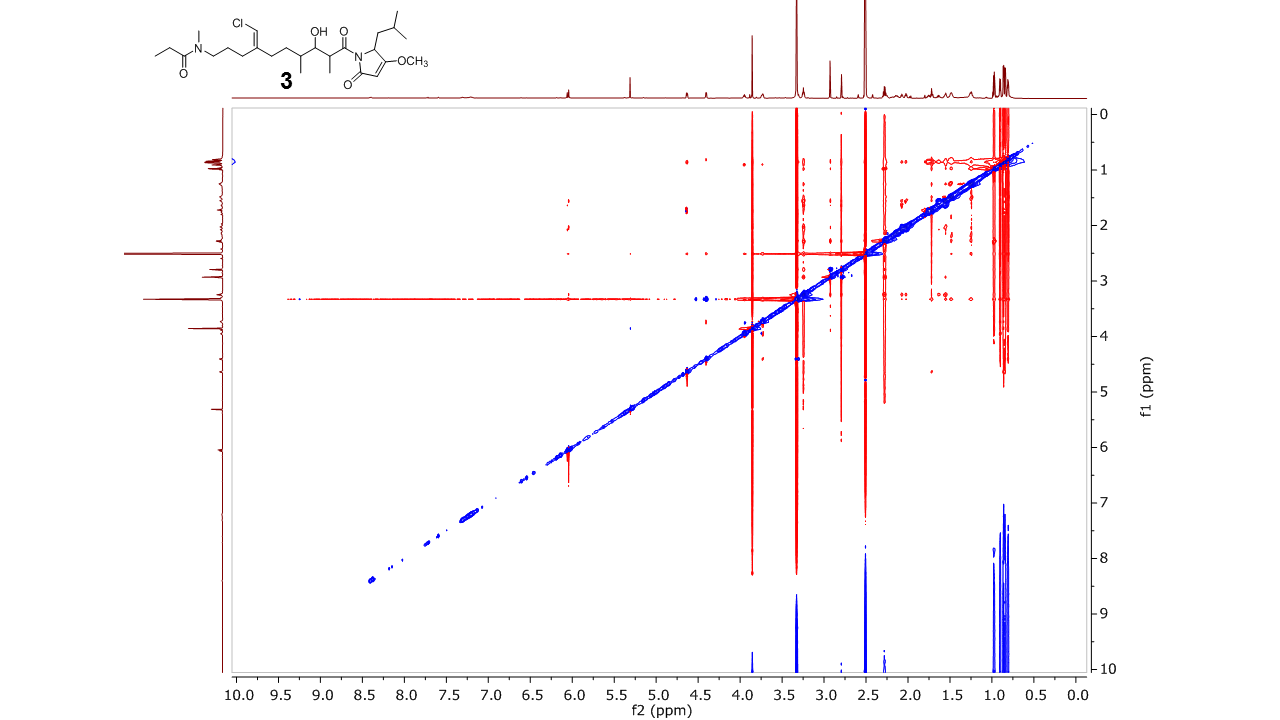
**

**Figure S28.** NOESY of **3**.

**
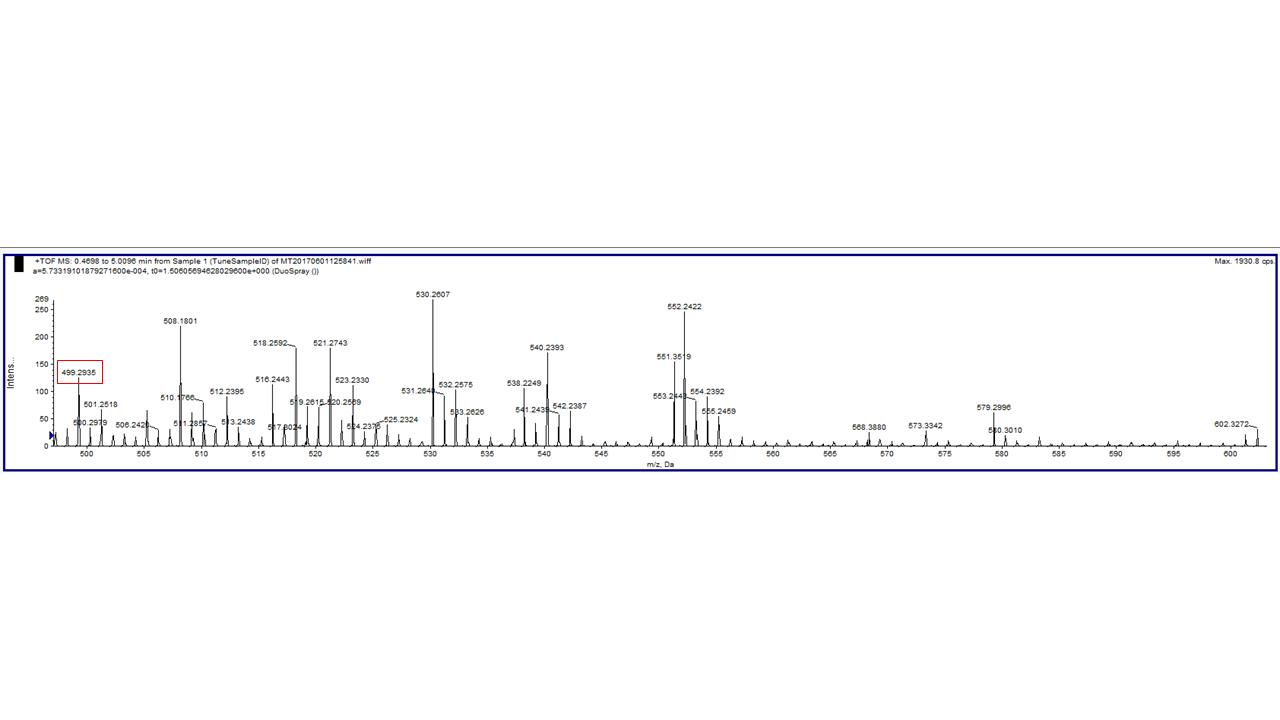
**

**Figure S29.** HRESIMS of **3**.

**
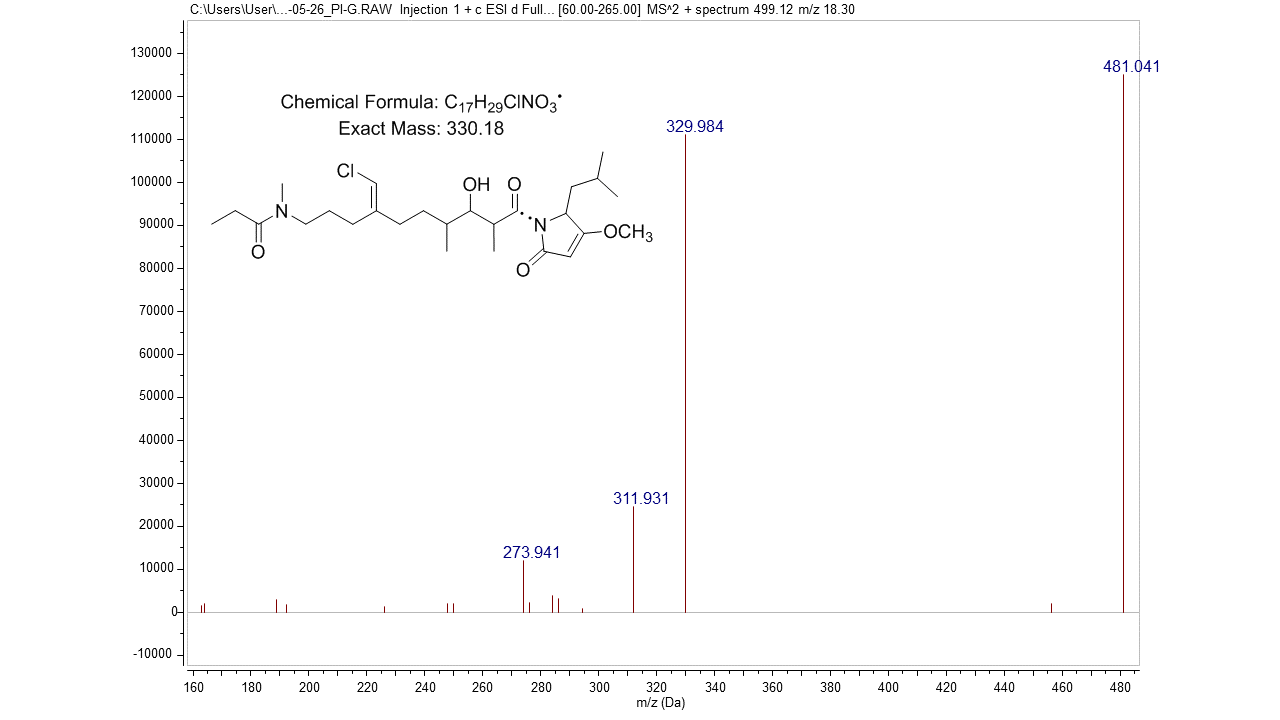
**

**Figure S30.** MS/MS spectrum of **3**.

**
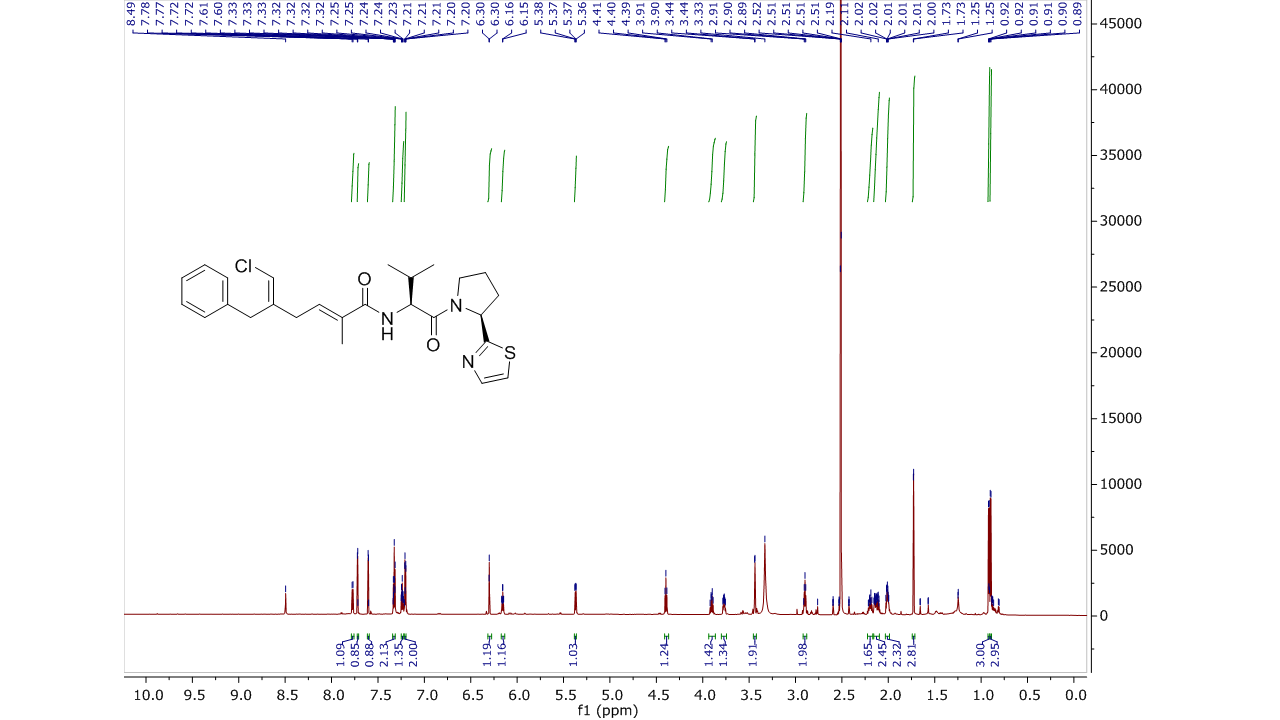
**

**Figure S31.** ^1^H NMR of smenothiazole A (800 MHz, DMSO).

**
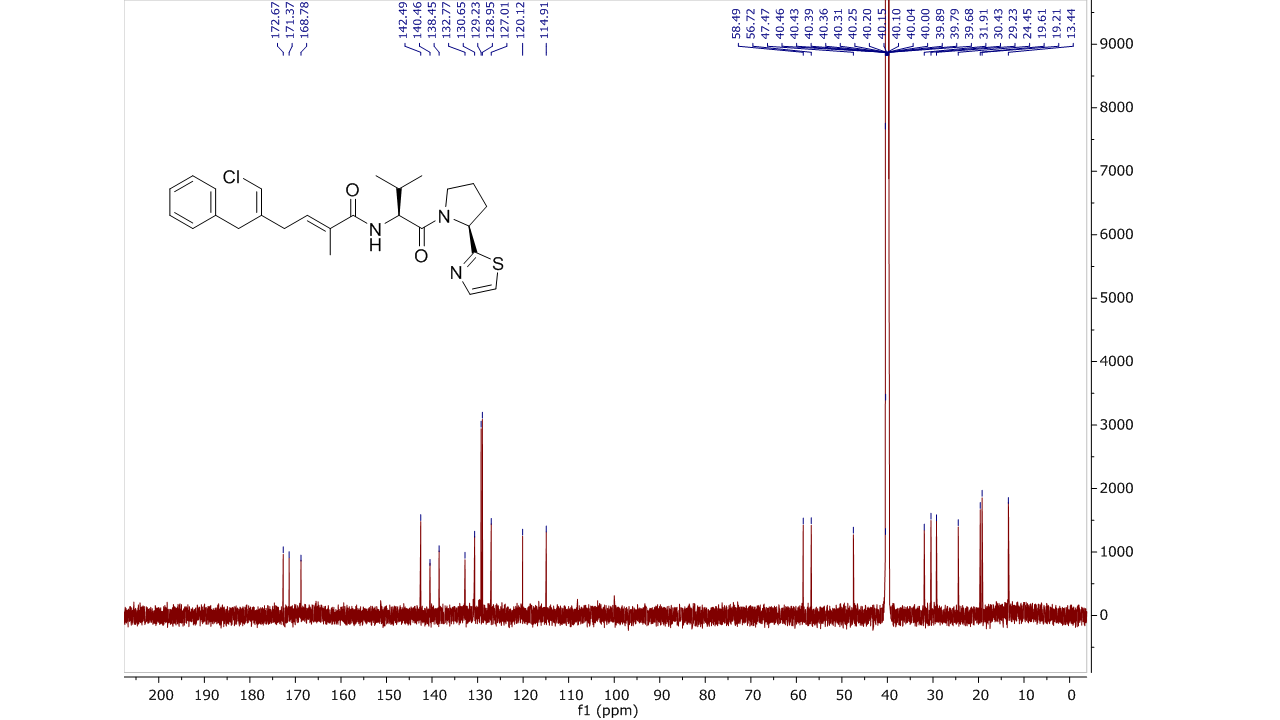
**

**Figure S32.** ^13^C NMR of smenothiazole A (200 MHz, DMSO).

**
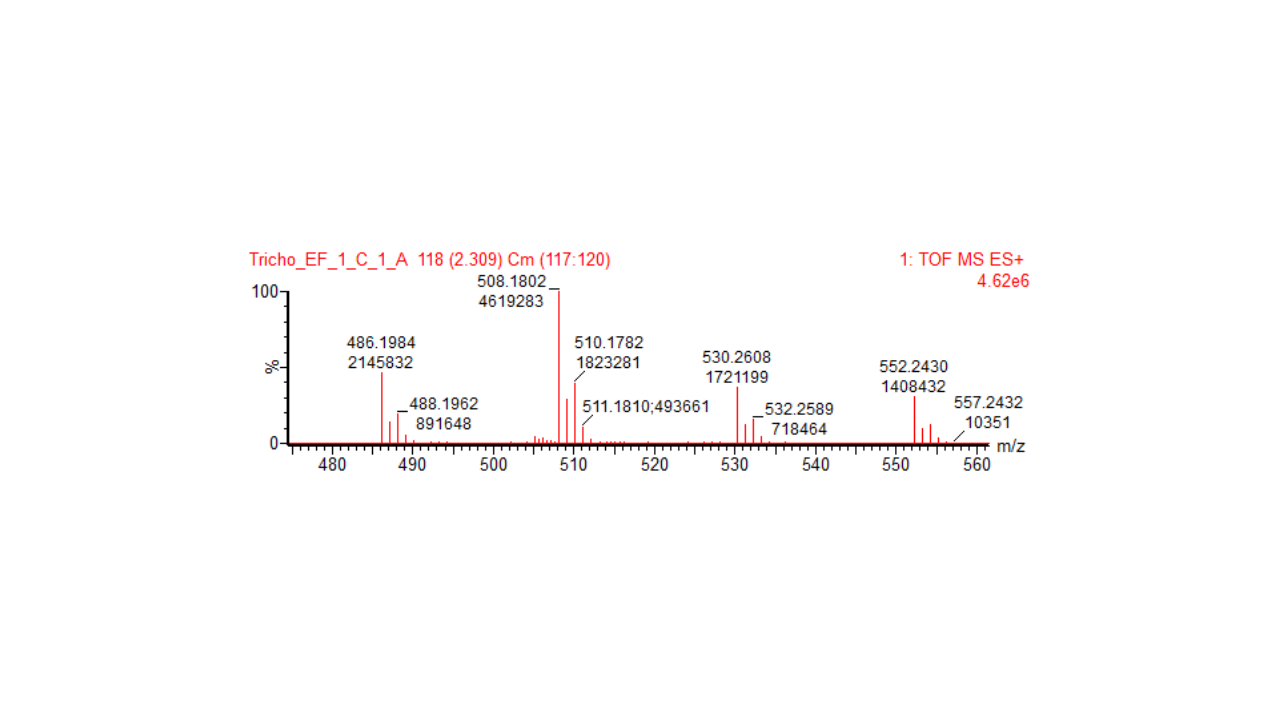
**

**Figure S33.** HRESIMS of smenothiazole A.

**
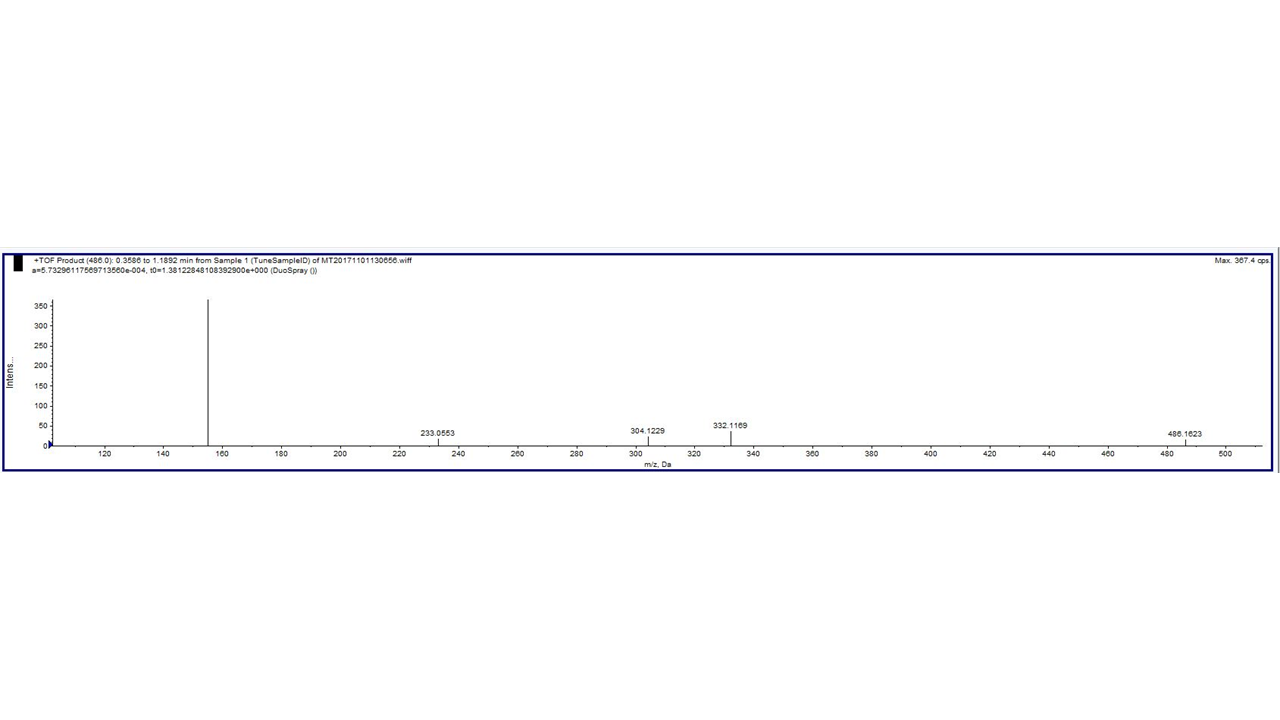
**

**Figure S34.** MS/MS spectrum of smenothiazole A.

**
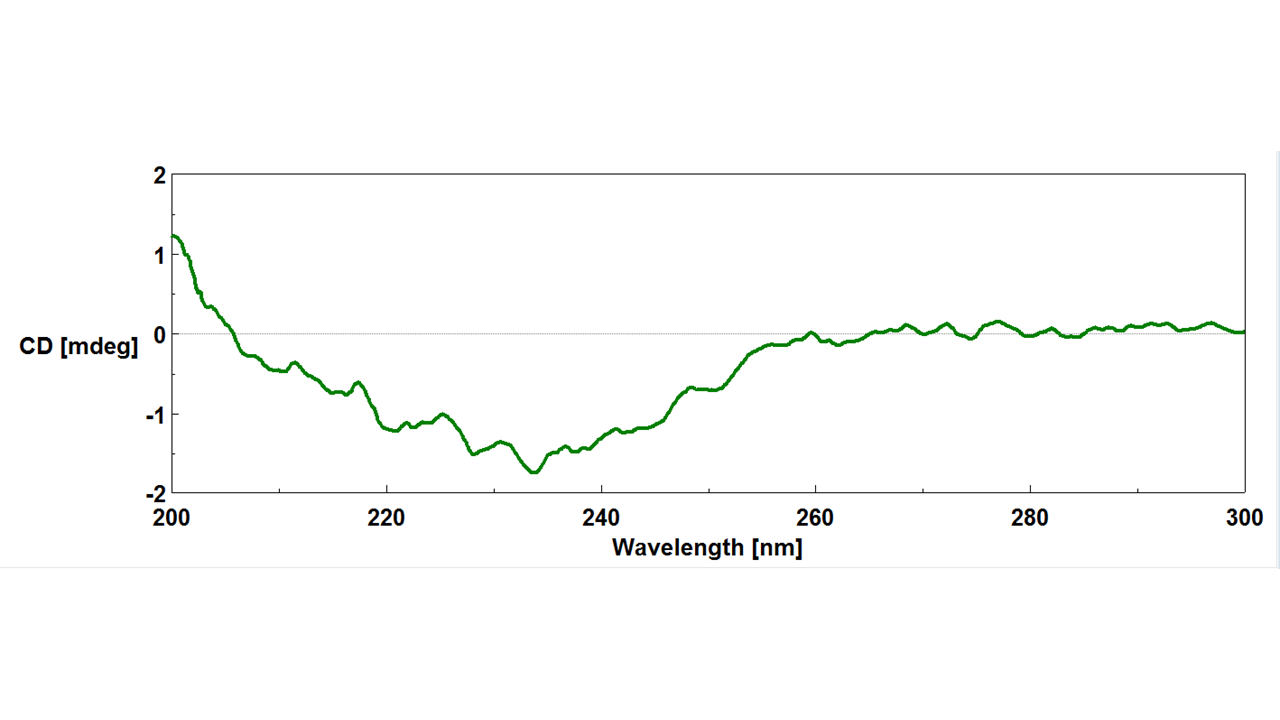
**

**Figure S35.** CD spectrum of smenothiazole A (CH_3_OH).

**
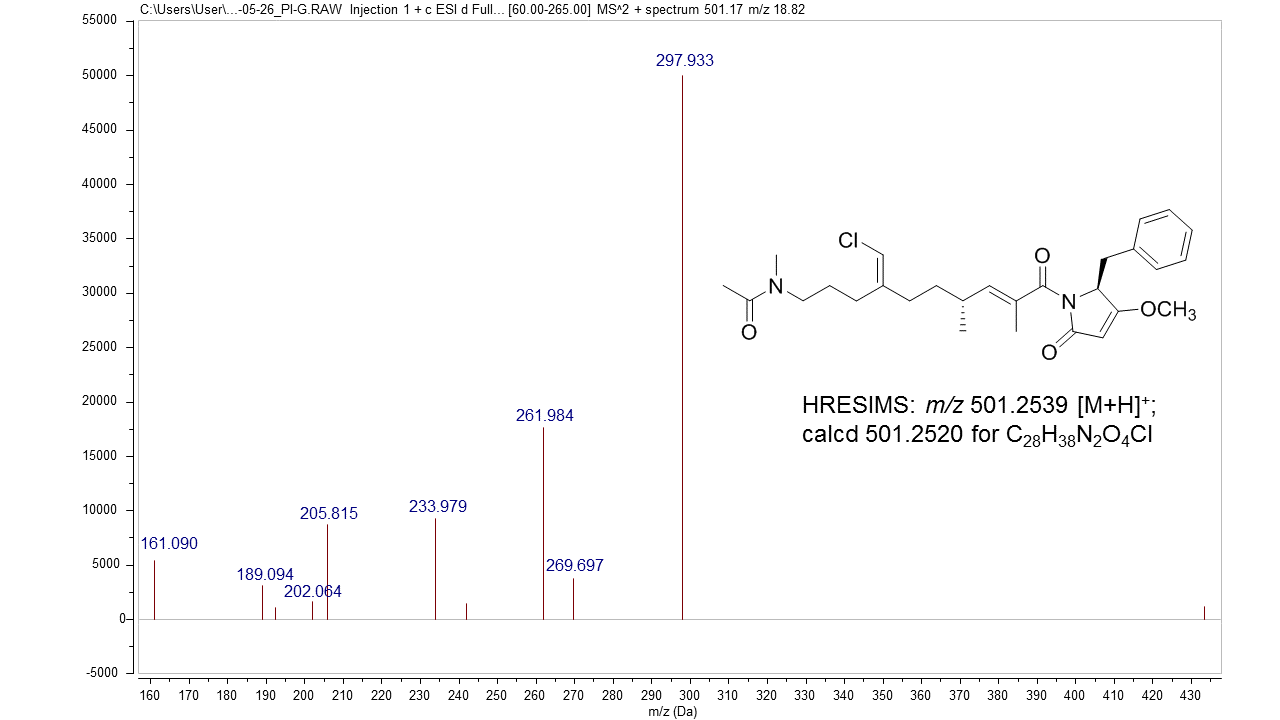
**

**Figure S36.** MS/MS of smenamide A/B detected in *Trichodesmium* chemical fractions.

**
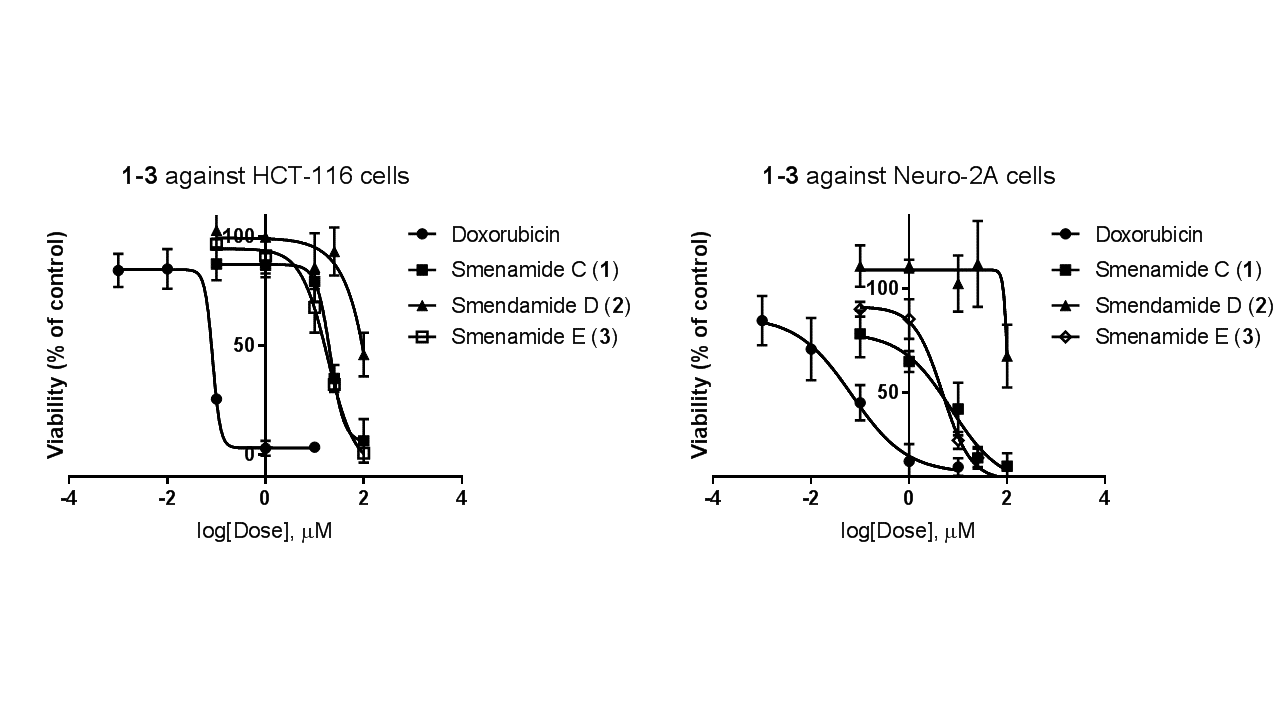
**

**Figure S37.** Dose-response curves of **1-3** against HCT-116 and neuro-2A cells.
